# 5’isomiR-183-5p|+2 elicits tumor suppressor activity in a negative feedback loop with E2F1

**DOI:** 10.1186/s13046-022-02380-8

**Published:** 2022-06-02

**Authors:** Xiaoya Li, Birgitta Elisabeth Michels, Oyku Ece Tosun, Janine Jung, Jolane Kappes, Susanne Ibing, Nishanth Belugali Nataraj, Shashwat Sahay, Martin Schneider, Angelika Wörner, Corinna Becki, Naveed Ishaque, Lars Feuerbach, Bernd Heßling, Dominic Helm, Rainer Will, Yosef Yarden, Karin Müller-Decker, Stefan Wiemann, Cindy Körner

**Affiliations:** 1 Division of Molecular Genome Analysis, German Cancer Research Center (DKFZ), Im Neuenheimer Feld 580, 69120 Heidelberg, Germany; 2 Medical Faculty Heidelberg, University of Heidelberg, Im Neuenheimer Feld 672, 69120 Heidelberg, Germany; 3 Current address: Neuroendocrine Tumor Center, the First Affiliated Hospital of Nanjing Medical University, Nanjing, China; 4 Faculty of Biosciences, University of Heidelberg, Im Neuenheimer Feld 234, 69120 Heidelberg, Germany; 5 Division of Applied Bioinformatics, German Cancer Research Center (DKFZ), Berliner Straße 41, 69120 Heidelberg, Germany; 6 Department of Biological Regulation, Weizmann Institute of Science, 76100 Rehovot, Israel; 7 Digital Health Center, Berlin Institute of Health (BIH) at Charité – Universitätsmedizin Berlin, Kapelle-Ufer 2, 10117 Berlin, Germany; 8 Genomics and Proteomics Core Facility, German Cancer Research Center (DKFZ), Im Neuenheimer Feld 280, 69120 Heidelberg, Germany; 9 Tumor Models Core Facility, German Cancer Research Center (DKFZ), Im Neuenheimer Feld 280, 69120 Heidelberg, Germany

**Keywords:** MicroRNAs, IsomiRs, MiR-183-5p, Cell cycle, E2F1, Triple-negative breast cancer

## Abstract

**Background:**

MicroRNAs (miRNAs) and isomiRs play important roles in tumorigenesis as essential regulators of gene expression. 5’isomiRs exhibit a shifted seed sequence compared to the canonical miRNA, resulting in different target spectra and thereby extending the phenotypic impact of the respective common pre-miRNA. However, for most miRNAs, expression and function of 5’isomiRs have not been studied in detail yet. Therefore, this study aims to investigate the functions of miRNAs and their 5’isomiRs.

**Methods:**

The expression of 5’isomiRs was assessed in The Cancer Genome Atlas (TCGA) breast cancer patient dataset. Phenotypic effects of miR-183 overexpression in triple-negative breast cancer (TNBC) cell lines were investigated *in vitro* and *in vivo* by quantifying migration, proliferation, tumor growth and metastasis. Direct targeting of *E2F1* by miR-183-5p|+2 was validated with a 3’UTR luciferase assay and linked to the phenotypes of isomiR overexpression.

**Results:**

TCGA breast cancer patient data indicated that three variants of miR-183-5p are highly expressed and upregulated, namely miR-183-5p|0, miR-183-5p|+1 and miR-183-5p|+2. However, TNBC cell lines displayed reduced proliferation and invasion upon overexpression of pre-miR-183. While invasion was reduced individually by all three isomiRs, proliferation and cell cycle progression were specifically inhibited by overexpression of miR-183-5p|+2. Proteomic analysis revealed reduced expression of E2F target genes upon overexpression of this isomiR, which could be attributed to direct targeting of *E2F1,* specifically by miR-183-5p|+2. Knockdown of *E2F1* partially phenocopied the effect of miR-183-5p|+2 overexpression on cell proliferation and cell cycle. Gene set enrichment analysis of TCGA and METABRIC patient data indicated that the activity of E2F strongly correlated with the expression of miR-183-5p, suggesting transcriptional regulation of the miRNA by a factor of the E2F family. Indeed, *in vitro,* expression of miR-183-5p was regulated by E2F1. Hence, miR-183-5p|+2 directly targeting *E2F1* appears to be part of a negative feedback loop potentially fine-tuning its activity.

**Conclusions:**

This study demonstrates that 5’isomiRs originating from the same arm of the same pre-miRNA (i.e. pre-miR-183-5p) may exhibit different functions and thereby collectively contribute to the same phenotype. Here, one of three isomiRs was shown to counteract expression of the pre-miRNA by negatively regulating a transcriptional activator (i.e. E2F1). We speculate that this might be part of a regulatory mechanism to prevent uncontrolled cell proliferation, which is disabled during cancer progression.

**Graphical Abstract:**

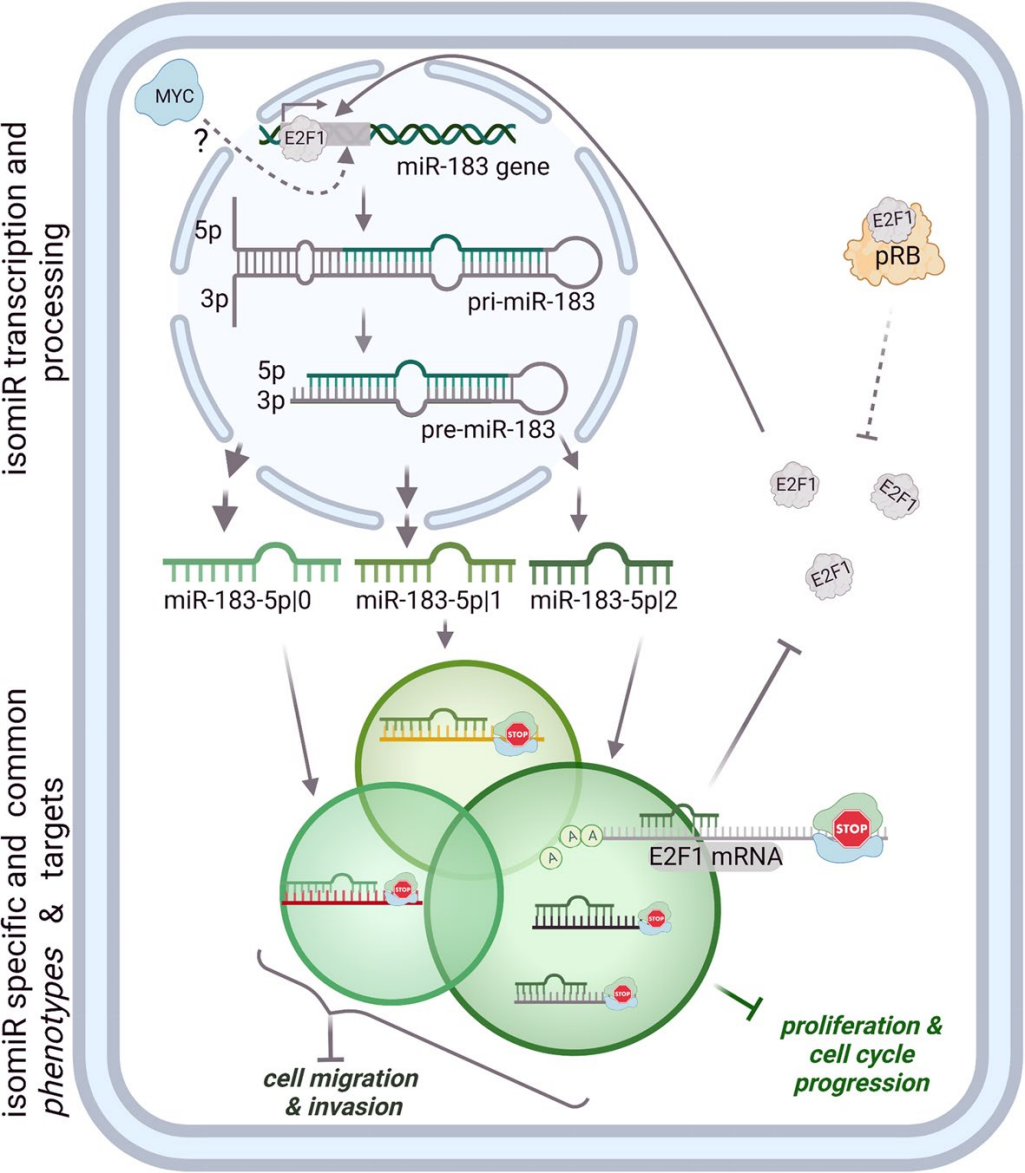

**Supplementary Information:**

The online version contains supplementary material available at 10.1186/s13046-022-02380-8.

## Background

Breast cancer is the most frequently diagnosed cancer type and the second leading cause of cancer death in females. According to the American Cancer Society’s statistics in 2021 [1] breast cancer is estimated to account for 30% of all new cancer cases and 15% of cancer-related death among women [2, 3]. Besides being a common condition, breast cancer is a complex and heterogeneous disease comprising of different subtypes based on molecular and clinical features [4]. These subtypes differ in their disease mechanisms, treatment options and in patient outcome, with triple-negative breast cancer (TNBC) being the most difficult to treat due to the lack of targeted therapy options [5, 6]. Therefore, a deeper understanding of the molecular mechanisms driving progression of TNBC is of crucial importance.

MicroRNAs (miRNAs) are short, single-stranded and non-coding RNA molecules that serve as post-transcriptional regulators in many biological processes [7–9]. The biogenesis of miRNAs starts with the transcription of the miRNA gene into a large primary transcript (pri-miRNA). Transcription of many miRNAs is positively or negatively regulated by transcription factors, such as p53, MYC, ZEB1 and ZEB2 [10]. The pri-miRNA is first cleaved into a precursor miRNA (pre-miRNA) [11] to then give rise to the mature miRNA which can arise from both strands of this short miRNA duplex [12]. As post-transcriptional regulatory molecules, mature miRNAs guide the silencing complex to target the 3’UTR of mRNA via the seed region, i.e., nucleotides 2 to 8 from the 5’end of every miRNA [13].

The advent of high-throughput RNA sequencing has enabled the comprehensive analysis of the miRNome, including the identification of different variants of the same miRNA. Such length and/or sequence variants of the same miRNA are termed isomiRs [14]. While most isomiRs are functionally redundant compared to their canonical counterparts, the 5’isomiRs exhibit a shifted 5’end and therefore a shifted seed sequence resulting in a different target spectrum. In consequence, 5’isomiRs are prone to exhibit vastly different functions compared to the respective canonical miRNA. In a previous study, we showed that the 5’isomiR of miR-140-3p specifically inhibits proliferation and migration by directly regulating targets that are distinct from those of the canonical miRNA [15].

MiRNAs are aberrantly expressed in cancer and have been shown to influence tumor behavior and progression [16]. Various studies have demonstrated that miRNAs, as proto-oncogenes and tumor suppressor genes, regulate gene expression by binding to the mRNA of target genes thereby causing RNA degradation or inhibition of translation, and affecting the functional status of breast cancer cells [17, 18]. However, 5’isomiRs have not been taken into consideration in most of these previous studies.

In this study, we identified three 5’isomiRs of miR-183-5p, which we found highly upregulated in breast cancer patient data from The Cancer Genome Atlas (TCGA). However, we found that all three 5’isomiRs of miR-183-5p exerted tumor suppressive functions in TNBC cell lines. Especially, miR-183-5p|+2 exhibited a stronger growth suppression by reducing proliferation and repressing cell cycle in TNBC cell lines. Interestingly, miR-183-5p|+2 decreased proliferation and arrested cells in G1 by directly targeting E2F1 while the expression of miR-183-5p was transcriptionally regulated by the activity of E2F1. Thus, miR-183-5p|+2 is part of a negative feedback loop by directly targeting and potentially regulating the activity of E2F1, which in turn functions as a transcriptional activator of miR-183 expression.

## Methods

Detailed descriptions of the methods can be found in Additional file 3.pdf.

### Cell culture

MDA-MB-231 (HTB-26), BT-549 (HTB-122), HCC-1806 (CRL-2335) and HEK293FT (PTA-5077) were obtained from ATCC (LGC Standard GmbH, Wesel, Germany). MDA-MB-231 and HCC-1806 were cultured in RPMI1640 medium supplemented with 10% FBS and 1% L-Glu. BT-549 was maintained in RPMI1640 medium supplemented with 10% FBS, 1% L-Glu and 0.1% Insulin. HEK293FT was cultured in DMEM supplemented with 10% FBS, 1% L-Glu, 1% NEAA and 1% Geneticin.

All parental cell lines were incubated at 37°C with 5% CO_2_, tested for potential mycoplasma contamination on a regular basis, and were positively authenticated prior to and in the end of the study (Multiplexion GmbH, Heidelberg, Germany).

### IsomiR annotation

**Fig. 1:**
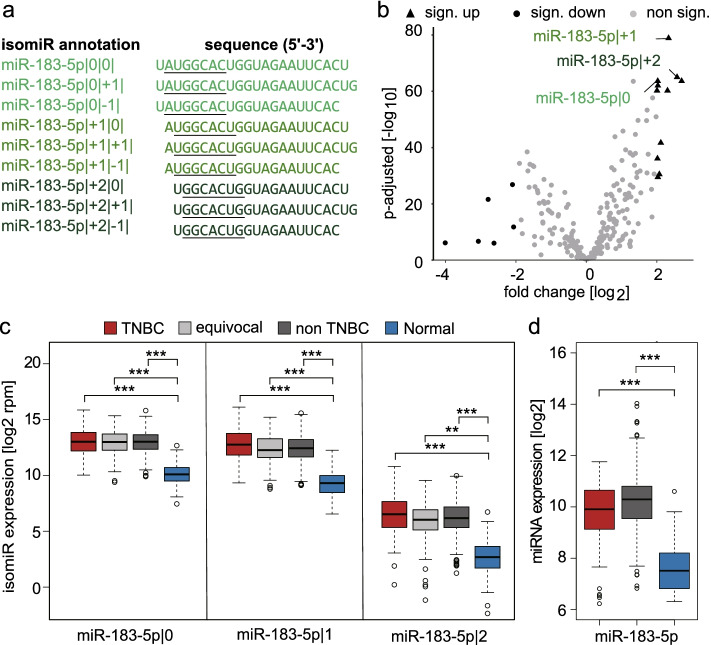
Three 5’isomiRs of miR-183-5p are highly upregulated in breast cancer. **a** The sequence of miR-183-5p and its isomiRs are presented. The seed sequences are underlined. Reads corresponding to isomiRs with identical 5’end depicted in the same color were summed up to obtain 5’isomiR expression. **b** Volcano plot of 294 isomiRs expressed >15 rpm in tumor and normal samples of breast cancer patients. Statistics: Unpaired two sample t-test, p-adjusted calculated using the “Benjamini and Hochberg”-method. IsomiRs with a fold change > 2 or < 2 and a p-adjusted < 0.05 are highlighted as sign. up and sign. down, respectively. **c** TCGA miRNA sequencing data from 1154 breast cancer patients were analyzed for the expression of three 5’isomiR-183-5p in different subtypes (TNBC: *n*= 177; Equivocal: *n*= 240; Non-TNBC: *n*= 689; Normal: *n*= 104). **d** METABRIC miRNA microarray data from 859 breast cancer patients were analyzed for the expression of miR-183-5p in different subtypes (TNBC: *n*= 102; Non-TNBC: *n*= 679; Normal: *n*= 78). (c-d) Statistics: ANOVA and Dunnett’s test, calculated in R. ** p-adjusted < 0.01, *** p-adjusted < 0.001

In this study, we focused on 5’isomiRs, neglecting the specific 3’ end of a miRNA molecule to reduce the complexity of the system and to focus on seed-specific effects. For the description of an isomiR, we employed the annotation introduced by Loher et al. [19]. Here, the position and direction of the sequence shift of an isomiR is annotated as exemplified in Fig. 1a. Specifically, miR-183-5p|0|0| represents the canonical miRNA with no shift at the 5’ or the 3’ end and has the following sequence: 5’-UAUGGCACUGGUAGAAUUCACU-3’. As we neglect the specific 3’ end of the sequence, the last three bases could be missing or the sequence could be extended by up to three additional bases to be considered miR-183-5p|0 in our analyses. Consequently, miR-183-5p|+1|0| has a 5’ end, which is shifted by one base, resulting in the following sequence: 5’-AUGGCACUGGUAGAAUUCACU-3’. miR-183-5p|+2|0| has this sequence: 5’-UGGCACUGGUAGAAUUCACU-3’. In the same way as detailed for miR-183-5p|0, reads differing by up to three nucleotides at the 3’ end of the sequence were considered as miR-183-5p|+1 and |+2, respectively.

For expression analysis in TCGA patient data, all reads with the same 5’ end were aggregated to focus on functional entities sharing the same seed sequence (Fig. 1a).

### Transfection

Cells were seeded at 70-80% confluency one day before transfection. All transfections (plasmids, siRNAs or miRNA mimics) were performed using Lipofectamine2000 (LF2000) according to the manufacturer’s instructions (0.4 μL/well in 96-well plates, 4 μL/well in 6well plates and 10 μL/dish in 10 cm dishes). Cells were incubated for 48 or 72 h at 37 °C, 5% CO_2_ in a humidified atmosphere before they were used for experiments.

All siRNAs (Dharmacon, Lafayette, USA) and synthesized miRNA mimics (Qiagen, Hilden, Germany) were used at a final concentration of 30 nM. The siRNAs purchased from siTOOLs Biotech (Planegg, Germany) were used at a final concentration of 2 nM. The sequences of siRNAs and miRNA mimics are listed in Additional file 2: Supplementary Table 1.

### Generation of stable cell lines

To generate TNBC cell lines overexpressing pre-miRNAs, the pre-miRNAs were cloned into a retroviral vector RT3GEPIR [20] and retroviral particles were produced in HEK293FT cells by co-transfection of the retroviral RT3GEPIR vector, together with the VSV.G envelope plasmid pMD2.G (Addgene #12259) and gag/pol packaging plasmid pHIT60 [21].

To generate inducible E2F1 overexpressing TNBC cell lines, the open reading frame of human *E2F1* (Addgene plasmid # 70329) was shuttled into the lentiviral expression vector rwSMART-TRE3G-GW-mCMV-TetON3G (Cellular Tools DKFZ, Heidelberg, Germany) using Gateway cloning technology [22] (ThermoFisher, Braunschweig, Germany). Lentiviral particles were produced in HEK293FT cells by co-transfection of the lentiviral rwSMART-TRE3G-E2F1-mCMV-TetON3G expression vector, together with 2^nd^ generation viral packaging plasmids VSV.G (Addgene #14888) and psPAX2 (Addgene #12260).

Virus-containing supernatant was collected 24 h after transfection. Centrifuged and filtered supernatant was used to transduce target cells in the presence of 10 μg/mL polybrene (Merck, Germany). 24 h after transduction, virus-containing medium was replaced with full growth medium containing 2 μg/mL puromycin.

### Xenograft assay

3x10^6^ MDA-MB-231 cells in 30 μL PBS:Matrigel (Corning, Bedford, USA, growth factor reduced, 1:1, v/v) stably overexpressing pre-miRNA (pre-miR-183 or two different pre-miRNA negative controls) were injected under isoflurane anesthesia into the 3^rd^ mammary gland fat pad of 6-7 week-old female NSG mice (n=6/group). One week after inoculation, doxycycline (1 mg/mL in drinking water supplemented with 5% saccharose) was given. The Kliba 3307 and the drinking water including ingredients were replaced once every week throughout the study. Twice a week, the tumor size was measured by caliper in two dimensions. The weight of mice was recorded once weekly. Mice were followed up for 12 weeks and sacrificed once the tumor reached 1 cm in one diameter or if an alternative predefined humane endpoint was reached. Lungs were collected and checked for micrometastasis by Alu PCR [23]. The animal experiment was licensed under G288/14 by local regulatory authorities.

### Transwell-based cell migration and invasion assay

Cells were starved in 0% FBS starvation medium for 24 h. Then, 100,000 cells in 200 μL of 0% FBS starvation medium were seeded into the upper compartment of Transwell inserts (Corning, Kaiserslautern, Germany). Full growth medium was used in the lower compartment as chemoattractant. Cells were allowed to migrate or invade for 16 h. In parallel, a black clear-bottom 96-well plate was prepared as a seeding control plate for normalization. The cells on the lower side of the membrane were fixed with 4% PFA for 15 min. Migrated cells and seeding control plate were stained with Hoechst 33342 and imaged with a Molecular Devices Microscope IXM XLS (Molecular Device, California, USA) using 4x S Fluor objective. Nuclei were defined by Hoechst signals within a certain size (6-35 μm) and intensity (5000 gray levels above local background) and counted using Molecular Devices Software (Molecular Device, California, USA). Afterwards, the exemplary membranes were stained with 0.5% crystal violet for 30 min and then imaged with a light microscope.

### Cell viability assay

Cell viability was analyzed with a microscope-based nuclei counting method. Briefly, cells were seeded into black clear-bottom 96-well plates and transfected 24 h after seeding using Lipofectamine 2000. At different time points, cell nuclei were stained with Hoechst 33342 for 30 min and propidium iodide (20 ng/well, Thermo Fischer Scientific, Massachusetts, USA) for 15 min. Subsequently, the plates were imaged with Molecular Devices Microscope IXM XLS (Molecular Devices, California, USA) using 4x S Fluor objective. The cell number was obtained by counting cell nuclei on each image. Nuclei were defined by Hoechst signals within a certain size (6-35 μm) and intensity (5000 gray levels above local background), counted and automatically classified for positivity in the propidium iodide channel with Molecular Devices Software (Molecular Device, California, USA). The mean value of six technical replicates was used for each biological replicate.

### BrdU/7ADD-based cell cycle assay

Cell cycle phases were analyzed with Bromodeoxyuridine (BrdU) and 7-Aminoactinomycin D (7-AAD) according to the manufacturer’s instructions. Briefly, cells were starved in 0% FBS starvation medium for 24 h, released from cell cycle block with full growth medium for 24 h and incubated with 10 μM BrdU 2 h prior to harvest. Cells were permeabilized by the Perm/Wash buffer and fixed with 250 μL Cytofix/Cytoperm buffer for 20 min at room temperature and incubated with 300μg/mL DNase for 1 h at 37°C. Cells were stained with Anti-BrdU antibody and 7-AAD and analyzed using a FACSCalibur device and CellQuest Pro (BD Biosciences, USA) and BD FACS DIVA software (BD Biosciences, USA).

### RNA extraction and qRT-PCR

RNA was isolated using the RNeasy or miRNeasy Kit (Qiagen, Hilden, Germany) according to the manufacturer’s recommendations. The concentration of total RNA was determined by NanoDrop ND-1000.

cDNA for mRNA analysis was prepared using the RevertAidTM H minus First-strand Kit (Thermo Fischer Scientific, Massachusetts, USA). Primers and probes are listed in Additional file 2: Supplementary Table 3. For quantification of miRNAs, miScript RT and PCR system (Qiagen, Hilden, Germany) was used. Raw data analysis was performed by using QuantStudio PCR Systems (Applied Biosystems). Data acquisition and raw data analysis were performed using QuantStudio PCR Systems (Applied Biosystems) with the ΔΔCt method [24].

### Protein isolation and Western blotting

Cells were seeded in 6-well plates for pre-treatment (miRNA mimics and siRNAs). After treatment, the cells were lysed with RIPA lysis buffer (Thermo Fisher Scientific, Massachusetts, USA) containing Complete Mini protease inhibitor cocktail and PhosSTOP phosphatase inhibitor (Roche Applied Science, Penzberg, Germany). The protein concentrations of the samples were determined by the BCA Protein Assays Kit (Thermo Fisher Scientific, Massachusetts, USA) and quantified with a GloMax microplate reader (Promega GmbH, Walldorf, Germany).

The primary and secondary antibodies used in this study are listed in Additional file 2: Supplementary Table 4. The membranes were scanned and probed using the Odyssey Infrared Imaging System (LI-COR Biosciences, Nebraska, USA). The signal intensity of the band was quantified by using ImageStudio software and median background subtraction (LI-COR Biosciences, Nebraska, USA).

### Mass spectrometry

Protein samples (10 μg per sample) were submitted to the DKFZ Genomics and Proteomics Core Facility for mass spectrometry-based protein analysis. Briefly, unfractionated samples were used for in-gel digestion on a DigestPro MSi robotic system (INTAVIS Bioanalytical Instruments) [25]. Peptides were separated on a cartridge trap column and eluting peptides were analyzed online by a coupled Q-Exactive-HF-X mass spectrometer (Thermo Fisher Scientific, Massachusetts, USA) running in the data depend acquisition mode.

Raw data was analyzed by the MaxQuant computational platform (version 1.6.3.3) using an organism-specific database extracted from Uniprot.org under default settings. Quantification was done by using a label-free quantification (LFQ) approach based on the MaxLFQ algorithm [26]. The Perseus software package (version 1.6.13.0) was used for imputation of missing values for GSEA analysis at default settings and for statistical analysis [27].

### Luciferase reporter assay

Direct targets of a miRNA of interest were validated by a 3’UTR dual luciferase reporter assay. The 3’UTR of *E2F1* was cloned into psiCHECK-2 and subjected to site-directed mutagenesis of the predicted seed match for miR-183-5p|+2. The sequences of primers for cloning are listed in Additional file 2: Supplementary Table 2.

1.2x10^4^ cells were seeded in white 96-well plates and transfected with miRNA mimics and 3’UTR reporter plasmid. 48 h after transfection, cells were washed and lysed and a dual luciferase assay was performed using a GloMax Microplate Reader (Promega Gmbh, Walldorf, Germany). The compositions of the buffers used in the luciferase assay are listed in Additional file 2: Supplementary Table 5.

### miRNA target prediction

The 3’UTR sequences for the expressed genes were extracted using GEO dataset GSE27003 [28]. Reads from cell lines BT-20, BT-474, MCF7, MDA-MB-231, MDA-MB-468, T-47D, ZR-75-1 were aligned using the STAR (version 2.7.3a) algorithm [29] to the hg38 genome assembly and gencode v22 gene model. Reads aligning to 3’UTR regions of a gene were merged to get the expressed 3’UTR sequence. Non-overlapping regions found in the same UTR definition as provided by the gencode models were considered separately.

MiR-183-5p and its 5’isomiRs were subjected to target prediction by using miRanda (version 3.3a) [30] and Targetscan (version 7.1) [31] algorithms to the expressed 3’UTR sequences. A consensus set of miRNA-targets, i.e. an overlap of transcript/miRNA pair between the two prediction algorithms, was computed based on the principle of complementing algorithms as described by Riffo-Campos et al [32]. Venn diagrams visualizing the overlap between the target predictions for different isomiRs were created using an online tool (http://bioinformatics.psb.ugent.be/webtools/Venn/).

### TCGA and METABRIC patient data analysis

Breast Cancer (BRCA) expression quantification data for mRNA and miRNA were obtained from The Cancer Genome Atlas (TCGA) Research Network (https://www.cancer.gov/tcga). mRNA gene expression data (FPKM-UQ) were downloaded from the Genomic Data Commons (GDC) harmonized database (https://portal.gdc.cancer.gov/projects/TCGA-BRCA) using the Bioconductor R package TCGAbiolinks (version 2.12.6) and the GRCh38 build (hg38). Batch corrected isomiR expression data was obtained from GEO dataset GSE164767 [33]. All isomiR reads with an identical 5’ position resulting in an identical seed sequence were collapsed, i.e. summed up, to only further investigate 5’isomiR variants and isomiRs with a median expression of >15 reads per million (RPM) were considered for analysis. Only data from patients with corresponding mRNA expression and miRNA expression data were considered for further analysis. TNBC classifications were derived from Lehmann et al. and Koboldt et al. ([5, 34]). Patients classified as negative for ER, PR and HER2 receptors in the study by Koboldt et al. were labelled as TNBC patients. Patients lacking information for one of the three receptors were labelled ‘equivocal’. In case of clear labelling as TNBC in the Lehmann et al. study; ‘equivocal’ patents were re-labelled as TNBC. mRNA and miRNA expression data as well as patient information containing TNBC status from the METABRIC study were downloaded from https://ega-archive.org/dacs/EGAC00001000484/ and https://ega-archive.org/studies/EGAS00000000122 [35, 36].

To compare differences in isomiR expression in levels in the TCGA data between tumor and normal tissue, average expression in tumor tissue was divided by expression in matched non-tumor/normal tissue for all patients and the results were log2 transformed and the p-adjusted values were computed from an unpaired, two sample t-test (adjustment by the Benjamini-Hochberg method). isomiRs with a log2 fold change > 2 or < -2 and a p-adjusted < 0.05 were considered significant.

### E2F activity score

E2F activity scores were calculated from the TCGA-BRCA and METABRIC mRNA data as follows: Genes present in the MSigDB Hallmark E2F target gene signature (version v7.3) were used. Expression of each gene was z-scaled over all patients. The median values of these z-scaled expression values were calculated for each patient over all genes of the E2F target gene signature; this median value of z-scaled expression of E2F target genes was used as the “E2F activity score” for each patient. Associations between E2F activity scores and expression of 5’isomiR-183-5p for each patient were depicted as scatter plots, and correlation coefficients were calculated using Spearman’s correlation test.

### Gene set enrichment analysis

Gene set enrichment analysis (GSEA) was applied to the mass spectrometry data. Briefly, missing values of mass spectrometry data were imputed and log2 fold changes of protein expression were computed for every protein and for all three 5’isomiR-183-5p compared to the control group. Protein identifiers were linked to the encoding genes for further downstream analyses. Gene set enrichment analysis was performed on the pre-ranked gene list using the Hallmark Gene Set Collection (version 7.3), a weighted enrichment statistic and default parameters of GSEA software (version 4.1.0) [37–39]. The analyses were summarized in a bubble heatmap depicting the normalized enrichment score (NES) and the false discovery rate (q-value).

Gene set enrichment analysis of TNBC patients from TCGA and METABRIC datasets was performed to investigate the correlation between isomiR expression and the activity of the E2F and other pathways in these patients, again using the Hallmark Gene Set Collection (version 7.2) [39]. To address this, batch-corrected expression of the respective isomiR (TCGA data) or microarray-based expression values for miR-183-5p (METABRIC data) were converted to ranks across patients and used as parameter. Ranked mRNA expression data were used as an input file. Thereby, Spearman correlation coefficients between isomiR/miR and genes were used as a ranking metric and 1000 permutations were performed by phenotype to assess statistical significance. The results were visualized in a bubble heatmap depicting the normalized enrichment score (NES) and the false discovery rate (q-value).

To show individual GSEA graphs, we used an adapted version of the replotGSEA function from the Rtoolbox (https://github.com/PeeperLab/Rtoolbox) to re-arrange the output of the GSEA tool.

### Graphical illustration and statistical analysis

For analyses performed in R, we used R version 4.0.2 or 4.0.3 (R Core Team 2020. R: A language and environment for statistical computing. R Foundation for Statistical Computing, Vienna, Austria. URL https://www.R-project.org/) and RStudio (RStudio Team, 2020. RStudio: Integrated Development Environment for R. RStudio, PBC, Boston, MA. URL http://www.rstudio.com/). Graphs were either generated using base R or using ggplot2 (version 3.3.3) [40] if not stated differently. Spearman correlations for comparison of different parameters in scatter plots were calculated in R.

For heatmap visualization of the non-imputed, z-scaled mass spectrometry results, the pheatmap package (version 1.0.12) [41] was used. Distance measures between rows were calculated by the Pearson correlation coefficient and clusters were compared by complete linkage.

If not mentioned differently, data are presented as mean ± SD. Statistical analysis was performed by a two-tailed Student’s t-test, Dunnet’s or Tukey’s multiple comparison test as indicated in the figures using GraphPad Prism (version 9.3.0) and p-values < 0.05 were considered statistically significant. *p*-value < 0.05, < 0.01 and < 0.001 are indicated with one, two and three asterisks respectively.

For the Fisher’s exact test on the Mass Spectrometry data, two gene sets (GOBP_NEGATIVE_REGULATION_OF_CELL_CYCLE_G1_S_PHASE_TRANSITION and GOBP_POSITIVE_REGULATION_OF_CELL_CYCLE_G1_S_PHASE_TRANSITION) were obtained from the Gene Ontology database [42]. We used the non-imputed mass spec results for this analysis (Additional file 2: Supplementary Table 9, lower table). Proteins were considered significantly regulated if a significant difference between control and the respective isomiR condition was observed in the Student’s T-test (p<0.05, performed by Perseus). Significantly up-or downregulated proteins required a log2 fold change between control and isomiR of >0 or <0, respectively. Using the proteins determined as upregulated or downregulated by these criteria as regulated proteins and all proteins detected in at least one sample in the Mass Spectrometry, significant enrichment of GO terms was calculated by Fisher’s exact test.

## Results

### Three 5’isomiRs of miR-183-5p are highly upregulated in breast cancer

Various previous studies have demonstrated the importance of miRNAs as biomarkers or drivers in cancer [43, 44]. However, these reports are mostly limited to investigations of canonical miRNAs, neglecting the functional relevance especially of 5’isomiRs. Therefore, we here aimed to characterize the 5’isomiR expression patterns in TCGA breast cancer patients. We employed the isomiR annotation introduced by Loher et al. [19] where the position and direction of the sequence shift of an isomiR is annotated as exemplified in Fig. 1a. Since 5’isomiRs frequently exhibit functional differences from their canonical counterpart due to a shift in the target-selective seed sequence, we focused on variants with altered 5’ends, neglecting the specific 3’end of an isomiR. For that purpose, all reads with the same 5’end were aggregated to focus on functional entities sharing the same seed sequence (Fig. 1a). To identify miRNAs and their 5’isomiRs with potential tumor-relevance, we analyzed miRNA sequencing data from the TCGA breast cancer dataset. We analyzed the differential expression between tumor and normal tissue samples for 294 5’isomiRs with an average expression of >15 RPM (Fig. 1b, Additional file 2: Supplementary Table 6). Out of these, 11 5’isomiRs were significantly upregulated in tumor tissue (black triangles, log2 fold change > 2, p-adjusted < 0.05) and six 5’isomiRs were significantly downregulated (black dots, log2 fold change < -2, p-adjusted < 0.05). Of note, all three 5’isomiRs of miR-183-5p were among the most significantly and most highly upregulated in tumor. The expression of the three 5’isomiRs of miR-183-5p was upregulated both, in triple-negative breast cancer and other subtypes compared to normal tissue in the TCGA dataset (Fig. 1c). Similarly, the expression of miR-183-5p was also significantly upregulated in both, TNBC and non-TNBC tumors as compared to benign breast tissue in the METABRIC cohort (Fig 1d).

### Pre-miR-183 exhibits tumor-suppressive phenotypes in TNBC cell lines

**Fig. 2:**
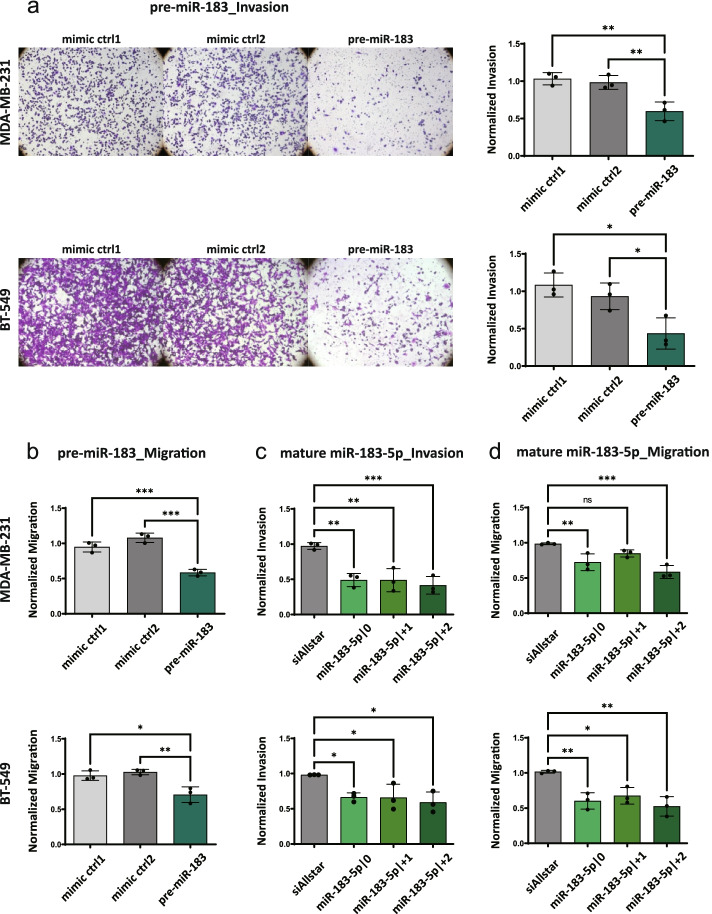
Pre-miR-183 exhibits tumor-suppressive phenotypes in TNBC cell lines. **a**-**b** Effect of pre-miR-183 overexpression on cell invasion and migration. pre-miR-183 overexpression was induced with doxycycline for 72 h in MDA-MB-231 and BT-549 cells. Cells were starved with 0% FBS medium for 24 h, seeded in starvation medium into transwell inserts (a) with or (b) without matrigel and were then allowed to invade towards 10% FBS as chemoattractant for 16 h. The cells on the lower side of the membrane were imaged and quantified with Molecular Devices Microscope IXM XLS. The number of migrated or invaded cells was normalized to the seeding control. Invaded cells were counterstained with crystal violet for visualization. Statistical analysis: ordinary one-way ANOVA followed by Tukey’s multiple comparisons test. **c-d** Effect of 5’isomiR-183-5p overexpression on cell migration and invasion. MDA-MB-231 and BT-549 cells were transfected with miRNA mimics or miRNA negative control for 48 h and then starved with 0% FBS medium for 24 h. The starved cells were reseeded in starvation medium into transwell inserts (c) with or (d) without matrigel and allowed to migrate or invade upon 10% FBS as chemoattractant for 16 h. The cells on the lower side of the membrane were imaged and quantified with Molecular Devices Microscope IXM XLS. The number of migrated or invaded cells was normalized to the seeding control. Values are depicted relative to the miRNA negative control. Data are presented as mean ± SD, *n* = 3 (each derived from the median of 3 technical replicates). Statistical analysis: ordinary one-way ANOVA followed by Dunnett’s multiple comparisons test *p < 0.05, **p < 0.01, ***p < 0.001 compared to control

Due to the upregulation of miR-183-5p and its 5’isomiRs, we hypothesized that the overexpression of these three 5’isomiR-183-5p would trigger tumor-promoting phenotypes. Since mature miRNAs and also isomiRs are generated from pre-miRNAs, TNBC cell lines with inducible overexpression of pre-miR-183 were generated to investigate the role of miR-183-5p and its 5’isomiRs. Expression of pre-miR-183 was increased in pre-miR-183 overexpressing MDA-MB-231 and BT-549 compared with two different pre-miRNA negative controls overexpressing TNBC cell lines as confirmed by miScript qRT-PCR (Additional file 2: Supplementary Table 7). Using these model systems, we firstly investigated the effect of pre-miR-183 on cell migration and invasion. In contrast to our initial hypothesis, cell invasion and migration were significantly reduced in both MDA-MB-231 and BT-549 cell lines stably overexpressing pre-miR-183 (Fig. 2a-b).

To dissect which isoforms were responsible for the reduction in cell migration and invasion caused by the overexpression of pre-miR-183, synthetic miRNA mimics were used to overexpress the individual isomiRs. In both TNBC cell lines, the overexpression of all three 5’isomiRs of miR-183-5p reduced cell migration and invasion compared to controls (Fig. 2c-d). To further confirm the reduction in cell migration and invasion *in vivo,* we injected MDA-MB-231 cells stably overexpressing pre-miR-183 into the mammary gland fat pad of NSG-mice and monitored primary tumor growth along with lymph node and lung metastasis. While three out of six mice in each control group had enlarged lymph nodes, no enlarged lymph nodes were detected in mice harboring tumors overexpressing pre-miR-183, indicating that indeed metastasis to the lymph nodes might be impaired. To further investigate metastasis to distant organs, DNA was isolated from the lungs of the mice and used for Alu qPCR to detect human DNA indicative of micro-metastases in the lungs. There was a trend of increased Ct values indicating decreased metastasis in mice harboring tumors overexpressing pre-miR-183. However, no significant difference in tumor metastasis to lung was observed in our experimental setup (Additional file 1: Supplementary Figure 1).

### MiR-183-5p|+2 is functionally different from the other two isoforms

**Fig. 3:**
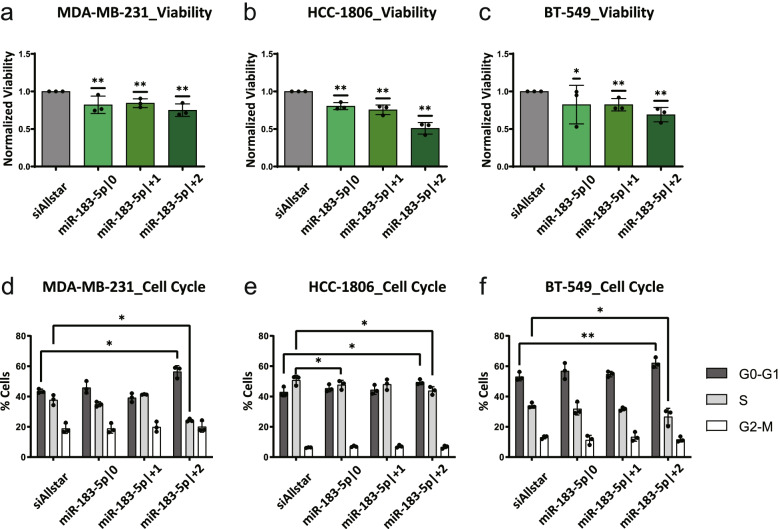
miR-183-5p|+2 is functionally different from the other two isoforms. **a**-**c** Effect of 5’isomiR-183-5p overexpression on cell viability. **a** MDA-MB-231, **b** HCC-1806 and **c** BT-549 cells were seeded into clear-bottom 96-well black plates and transfected with miRNA mimics or siAllstar. 72h after transfection, cells were stained with Hoechst 33342 and propidium iodide and quantified using Molecular Devices Microscope IXM XLS. The number of viable cells was measured by counting cell nuclei positive for Hoechst and negative for propidium iodide staining with Molecular Devices Software. Values were normalized to the respective control. Data are presented as mean ± SD, n = 3 (each derived from the median of 6 technical replicates). *p < 0.05, **p < 0.01, ***p < 0.001 compared to control, one sample t-test. **d-f** Effect of 5’isomiR-183-5p overexpression on cell cycle. **d** MDA-MB-231, **e** HCC-1806 and **f** BT-549 Cells were transfected with miRNA mimics or siAllstar for 48 h. After transfection, the cells were starved with 0% FBS medium for 24 h to partially synchronize the distribution of cell cycle, and subsequently were released with full growth medium for 24 h. Afterwards, the cells were pulsed with BrdU for 2 h to label cells actively synthesizing DNA during that time period. FITC conjugated anti-BrdU antibody was used to stain the cells with BrdU incorporation. 7-AAD, a dye binding to total DNA, was coupled with BrdU staining. With this combination, two-color flow cytometry analysis was applied to determine cell cycle distribution (G0-G1, S, and G2-M phase). Data are presented as mean ± SD, *n* = 3 (each derived from the median of 3 technical replicates). Statistical analysis: ordinary one-way ANOVA followed by Dunnett’s multiple comparisons test; *p < 0.05, **p < 0.01, ***p < 0.001 compared to control

While we could not conclusively confirm a role of pre-miR-183 in metastasis in our descriptive set-up of the mouse experiment, we observed a trend towards reduced tumor growth upon pre-miR-183 overexpression as indicated by reduced tumor volumes 33 days after injection (Additional file 1: Supplementary Figure 1b and c). This was also underlined by a trend of prolonged survival, i.e. the time to pre-defined humane endpoints, in the pre-miR-183 overexpression group, compared to the controls (Additional file 1: Supplementary Figure 1d). This difference was statistically significant when compared to control 1, but not compared to control 2. Yet, we decided to validate a potential role of pre-miR-183 in tumor cell proliferation. For that purpose, we next investigated the impact of synthetic miRNA mimics on cell viability and cell cycle *in vitro*. A microscopy-based cell counting assay was utilized to unravel the role of the three 5’isomiRs of miR-183-5p on cell proliferation in three TNBC cell lines, namely MDA-MB-231, HCC-1806 and BT-549. Of note, the overexpression of miR-183-5p|+2 strongly reduced the number of viable cells compared to siAllstar as miRNA mimics negative control for all tested cell lines (Fig. 3a-c). The other two 5’isomiRs, miR-183-5p|0 and miR-183-5p|+1, had slightly milder effects on cell viability. Mechanistically, this was partly caused by increased rates of cell death triggered especially by miR-183-5p|+2. Specifically, we investigated the percentage of cells taking up propidium iodide which is considered as a sign of disintegration of the cytoplasmic membrane during cell death. Here, we observed that cells overexpressing miR-183-5p|+2 displayed a 1.5-fold (MDA-MB-231) to 3-fold (HCC-1806) increase in the percentage of propidium iodide positive cells compared to control transfection (Additional file 1: Supplementary Figure 2 a-c). This effect was less prominent upon overexpression of the other two isomiRs of miR.183-5p. Next, to investigate the effect of all isoforms on the ability of cells to re-enter cell cycle after starvation-induced arrest in G1-phase, BrdU/7AAD staining and subsequent FACS analysis were performed 24 h after release from starvation. In all three TNBC cell lines, the overexpression of miR-183-5p|+2 arrested cells in G0/G1-phase compared to siAllstar and to the other two isoforms. At the same time, the cells in S-phase (BrdU-positive) were significantly decreased by miR-183-5p|+2 overexpression in all cell lines in comparison to the other two 5’isomiR-183-5p and negative control (Fig. 3d-f), indicating an impaired capability to overcome starvation-induced cell cycle arrest. In addition, S-phase was significantly reduced by miR-183-5p|0 in HCC-1806 cells. The results of the statistical comparison of all isomiRs to the control by Dunnett's multiple comparisons test and comparison of all conditions against each other by Tukey's multiple comparisons test are summarized in Additional file 2: Supplementary Table 8. Briefly, S-phase is significantly reduced by miR-183-5p|+2 compared to the other isomiRs in MDA-MB-231 cells, whereas these differences do not reach significance in BT-549 cells. In summary, among the investigated 5’isomiRs, miR-183-5p|+2 displayed the strongest inhibitory effect on cell cycle progression and cell viability in three genetically diverse TNBC models.

### Proteins encoded by E2F target genes are depleted in cells overexpressing miR-183-5p|+2

Having observed a strong reduction in cell proliferation and cell cycle progression specifically upon overexpression of miR-183-5p|+2, we next aimed to unveil the molecular mechanism and the associated specific direct targets underlying this phenotype. Hence, we hypothesized that miR-183-5p|+2 would regulate specific proteins or signaling pathways resulting in the differential regulation of growth-associated phenotypes. To address this hypothesis, label-free mass spectrometry was performed to examine alterations in the proteome upon overexpression of each isoform in an unbiased way. We reasoned that a protein-level profiling would be most informative as miRNAs exert their function at the post-transcriptional level and do not always affect the abundance of target mRNAs.

**Fig. 4:**
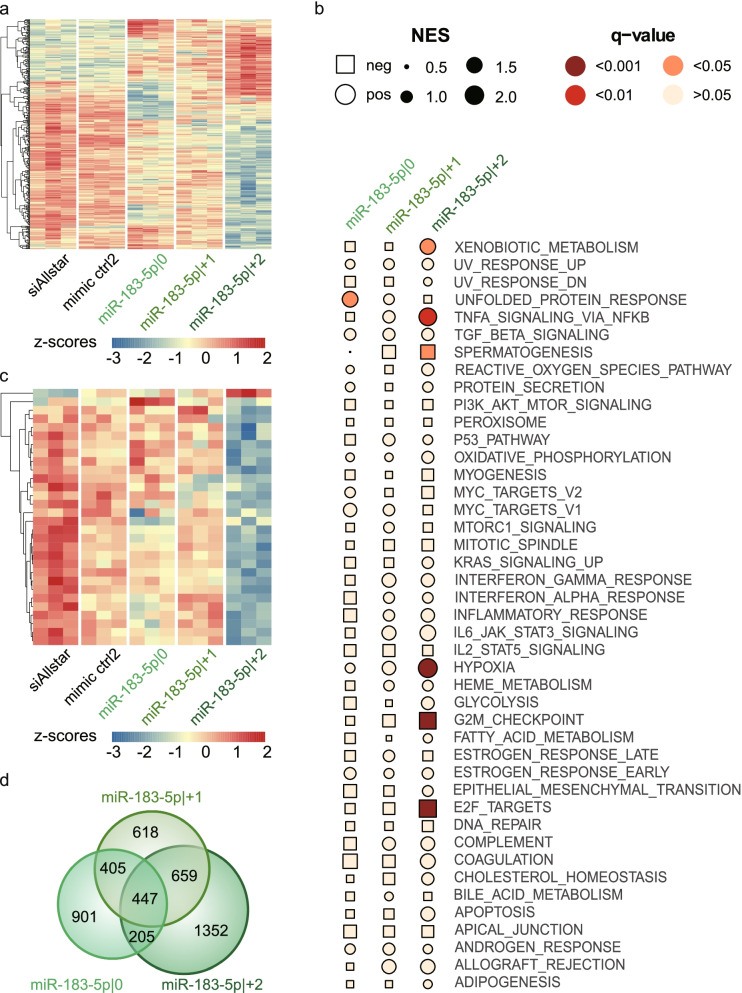
E2F target signature is depleted in cells overexpressing miR-183-5p|+2. **a** Mass spectrometry derived data from MDA-MB-231 cells overexpressing one of the 5’isomiRs of miR-183-5p were compared for differential protein expression (Student’s T-Test). Non-imputed, z-scaled expression of proteins with significantly different expression between samples are visualized as heatmap. **b** Gene set enrichment analysis was performed on the imputed proteome data for each of the overexpression cell lines using preranked GSEA and log2 foldchange as ranking metric. Normalized enrichment score (NES) and adjusted p-value of the respective gene set was used to visualize results in a bubble heatmap. **c** Heatmap showing z-scaled protein expression of all proteins within the gene set E2F targets for the samples overexpressing one of the isomiRs of miR-183-5p. Non-imputed proteome data were used. **d** Venn diagram showing the overlap between target prediction results of miR-183-5p and its 5’isomiRs. Consensus predictions of custom target predictions using both Miranda and TargetScan prediction algorithms were used

In total, 5864 proteins were detected, and 3690 of them were detected in all samples. For a general understanding, we first focused on proteins detected in all samples and significantly deregulated by one of the isomiRs as compared to the controls (355 proteins, Fig. 4a, Additional file 2: Supplementary Table 9). Of note, protein expression levels upon overexpression of miR-183-5p|+2 were clearly distinct in comparison to the other two isoforms and miRNA negative controls.

In order to identify the molecular mechanisms potentially underlying the effect of each isomiR on cancer cell phenotypes, gene set enrichment analysis was performed using proteomic data (Additional file 2: Supplementary Table 10) and the Hallmark Gene Set Collection (h.all.v7.3) [39]. Here, we observed highly significant associations with the gene sets ‘G2/M Checkpoint’, ‘Hypoxia’ and ‘E2F Targets’ (Fig. 4b, Additional file 2: Supplementary Table 11). The latter had the highest NES and was most significantly downregulated specifically by overexpression of miR-183-5p|+2. No such enrichment was observed with the other two isoforms (Additional file 1: Supplementary Figure 3). The specific downregulation of ‘E2F Targets’ by miR-183-5p|+2 was apparent also at the level of individual proteins compared to the other two isoforms or the negative controls (Fig. 4c, Additional file 2: Supplementary Table 12). This was further strengthend by a Fisher’s exact test using GO-term based genesets, namely GOBP_NEGATIVE_REGULATION_OF_CELL_CYCLE_G1_S_PHASE_TRANSITION and GOBP_POSITIVE_REGULATION_OF_CELL_CYCLE_G1_S_PHASE_TRANSITION to specifically test the hypothesis that the G1-arrest observed in our cell cycle analysis can also be confirmed on protein level specifically for miR-183-5p|+2. While we did not observe any significant association of miRNA overexpression with the positive regulation set, we confirmed a significant enrichment of negative regulators of G1-S transition within the set of proteins upregulated upon overexpression of miR-183-5p|+2. (Additional file 2: Supplementary Table 13). Together, the impaired ability of cells overexpressing miR-183-5p|+2 to re-enter cell cycle after starvation and the marked downregulation of a set of E2F target proteins in these cells let us hypothesize that there might be a mechanistic link.

E2Fs, a group of genes that encode a family of transcription factors (TFs), regulate G1/S transition in the cell cycle. All E2Fs are involved in cell cycle regulation, but only three of them, E2F transcription factor 1 (E2F1), E2F2 and E2F3a, are transcriptional activators. Hence, we hypothesized that miR-183-5p|+2 would directly target at least one of these activating TFs to achieve coordinated downregulation of downstream transcriptional targets.

Thus, for all three isomiRs of miR-183-5p, we predicted direct target sites within 3’UTR regions supported by breast cancer cell line RNA sequencing data to reduce the occurrence of false-positive predictions. To be more stringent, only consensus predictions from the TargetScan and miRanda3.3a algorithms were considered (Additional file 2: Supplementary Table 14). While there was a strong overlap in the predicted targets of each of the 5’isomiR-183-5p, also isomiR-specific targets were identified (Fig. 4d). Of note, the spectrum of uniquely predicted targets was the largest for miR-183-5p|+2. *E2F1* was among those genes that were specifically predicted as target of miR-183-5p|+2 with a seven base pairs binding site (7mer-m8; Additional file 1: Supplementary Figure 4). Therefore, we hypothesized that *E2F1* might be directly regulated by miR-183-5p|+2 and went on to test this experimentally.

### E2F1 is a direct target of miR-183-5p|+2

**Fig. 5:**
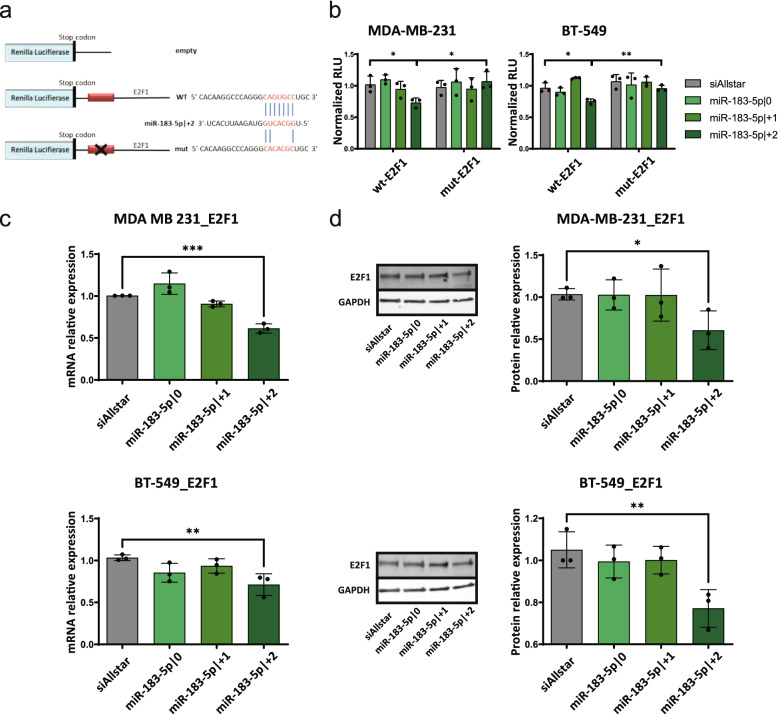
E2F1 is a direct target of miR-183-5p|+2. **a** Schematic representation of the different psiCHECK-2 constructs in 3’UTR reporter luciferase assay and the human *E2F1* mRNA indicating the binding site for miR-183-5p|+2. **b** Validation of direct targeting of E2F1 3’UTR by 3’UTR luciferase assay. MDA-MB-231 and BT-549 were seeded in white 96-well plates. psiCHECK-2, psiCHECK-2_E2F1_3’UTR or mutated psiCHECK-2_E2F1_3’UTR plasmid was cotransfected with miRNA mimics or miRNA negative control for 48 h. The cells were lysed and the activity of renilla and firefly luciferase was measured using GloMax Microplate Reader. Renilla luciferase measurements were normalized to the activity of firefly luciferase. The relative luciferase activity was normalized to the empty psiCHECK-2. Thereafter, values were normalized to miRNA negative control. **c-d** Downregulation of E2F1 by miR-183-5p|+2 overexpression at mRNA and protein level. MDA-MB-231 and BT-549 were seeded in 6-well plates and transfected with miRNA mimics (miR-183-5p|0, miR-183-5p|+1 and miR-183-5p|+2) or siAllstar (as miRNA negative control) for 48 h. **c** RNA was isolated using RNeasy kit (Qiagen). The mRNA expression levels of target genes were quantified by Taqman qRT-PCR. Gene expression was normalized to housekeeping genes (*ACTB* and *GAPDH*) using the ddCt method. Normalized gene expression is depicted as the relative expression compared to cells transfected with siAllstar. **d** Cells were lysed in RIPA buffer and protein expression level of E2F1 and GAPDH was determined by western blot. Representative blots are depicted. Band intensities were quantified using ImageStudio with median background subtraction method. The expression of E2F1 was normalized to GAPDH. The values are depicted as relative expression compared to cells transfected with siAllstar. Data are presented as mean ± SD, *n* = 3 (each derived from the median of 3 technical replicates). Statistical analysis: ordinary one-way ANOVA followed by Dunnett’s multiple comparisons test; *p < 0.05, ** *p* < 0.01, ***p < 0.001 compared to control

In order to validate direct targeting of *E2F1*, the full-length 3’UTR of *E2F1* was cloned into a dual luciferase reporter assay (Fig. 5a). To prove that the effect of miR-183-5p|+2 was indeed due to direct binding to the seed-matching sequences in the 3’UTR of *E2F1*, we mutated the seed region in the *E2F1* 3’UTR and tested whether the mutation in the miRNA-binding site would abrogate the downregulation by miR-183-5p|+2. As shown in Fig. 5b, the luciferase activity was specifically repressed by miR-183-5p|+2, while no decrease was observed upon overexpression of the other two 5’isomiR-183-5p. In addition, the significant downregulation of relative luciferase activity by miR-183-5p|+2 was completely rescued by the mutations of the seed-matching bases. Hence, the 3’UTR of *E2F1* was directly and specifically targeted by miR-183-5p|+2.

To validate the downregulation of *E2F1* by miR-183-5p|+2 at the mRNA and protein levels, MDA-MB-231 and BT-549 cells were transfected with miR-183-5p|0, miR-183-5p|+1, miR-183-5p|+2 mimics or a negative control. The *E2F1* mRNA level was significantly decreased upon miR-183-5p|+2 overexpression in both cell lines (Fig. 5c). Accordingly, E2F1 protein expression level was slightly but significantly reduced in both cell lines (Fig. 5d).

### Downregulation of E2F1 is partially responsible for the effect of overexpression of miR-183-5p|+2

**Fig. 6:**
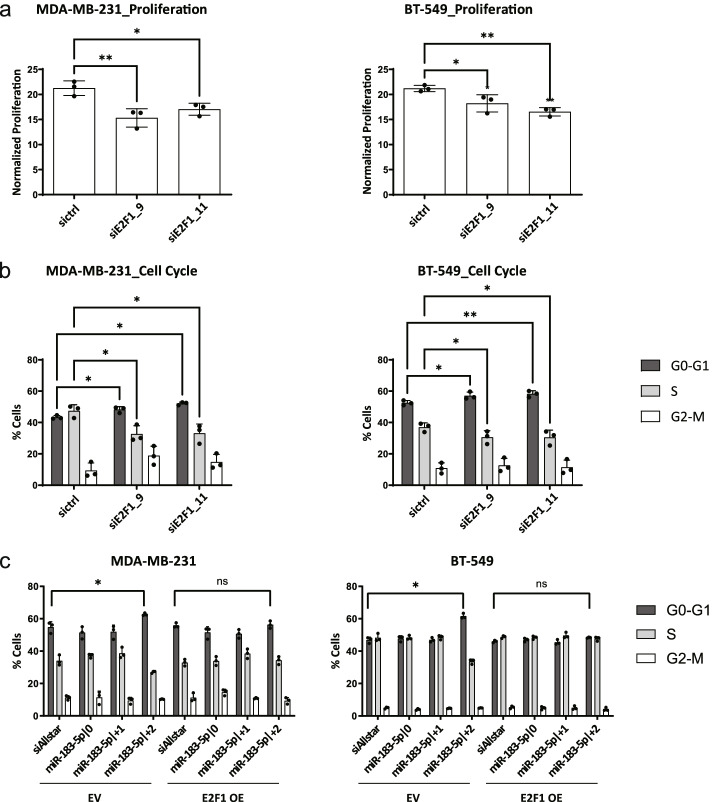
Targeting E2F1 partially causes the effect of overexpression of miR-183-5p|+2. **a** Effect of E2F1 knockdown on cell proliferation. MDA-MB-231 and BT-549 were transfected with siE2F1_9, siE2F1_11 or siRNA negative control for 48 h. After transfection, the cells were starved for 24 h and reseeded into clear-bottom 96-well black plates. Cells were stained with Hoechst 33342 for 30 min and imaged with Molecular Devices Microscope IXM XLS after attachment and 144 h later. The cell number was obtained by counting cell nuclei with Molecular Devices Software and normalized to the cell number at 0 h time point. Data are presented as mean ± SD, n = 3 (each derived from the median of 6 technical replicates). Statistical analysis: ordinary one-way ANOVA followed by Dunnett’s multiple comparisons test. **b** Effect of *E2F1* knockdown on cell cycle*.* After transfection, MDA-MB-231 and BT-549 cells were starved with 0% FBS medium for 24 h and released with full growth medium for 24 h. Afterwards, the cells were exposed to BrdU for 2 h and stained with FITC conjugated anti-BrdU antibody and 7-AAD. Cells cycle was analyzed using a FACSCalibur device and the CellQuest Pro software. Statistical analysis: two-way ANOVA followed by Dunnett’s multiple comparisons test. **c** Ectopic overexpression of *E2F1* ORF rescues the effect of miR-183-5p|+2 overexpression on cell cycle. MDA-MB-231 and BT-549 cells were seeded in 6-well plates and E2F1 expression was induced with the doxycycline. Cells were transfected with miRNA mimics (miR-183-5p|0, miR-183-5p|+1 and miR-183-5p|+2) or siAllstar for 48 h. After transfection, the cells were starved with 0% FBS medium for 24 h and released with full growth medium for 24 h. Cells were cultured in presence of doxycycline in each step (0.2 ng/mL for MDA-MB-231, 5 ng/mL for BT-549). Cells cycle was analyzed using a FACSCalibur device and the CellQuest Pro software. Data are presented as mean ± SD, *n* = 3 (each derived from the median of 3 technical replicates). *p < 0.05, **p < 0.01 compared to control, unpaired *t-*test

Having shown that miR-183-5p|+2 directly targets *E2F1*, we next hypothesized that the effect on cell proliferation and cell cycle upon overexpression of miR-183-5p|+2 was in part caused by downregulation of E2F1. To this end, a set of four different siRNAs targeting *E2F1* was used. Based on the knockdown efficiency of each individual *E2F1* siRNAs, we chose siE2F1_9 and siE2F1_11 for further study. The knockdown efficiency of siE2F1_9 and siE2F1_11 was confirmed (Additional file 1: Supplementary Figure 5) and, as anticipated, the knockdown of E2F1 resulted in a significant inhibition of cell proliferation in the MDA-MB-231 and BT-549 cell lines (Fig. 6a). The cells were arrested in G0/G1-phase upon transfection of siE2F1_9 or siE2F1_11 (Fig. 6b) and, in MDA-MB-231, S-phase was significantly decreased. In summary, downregulation of *E2F1* phenotypically resembles overexpression of miR-183-5p|+2 regarding cell proliferation and cell cycle in two TNBC cell lines indicating that this interaction might be partially responsible for the phenotype.

To further validate that miR-183-5p|+2 arrest cells in G0/G1-phase due to targeting within the 3’UTR of *E2F1*, we tested whether ectopic overexpression of the *E2F1* ORF (Additional file 1: Supplementary Figure 6) would rescue the effect of miR-183-5p|+2 overexpression on cell cycle. Indeed, the effect of miR-183-5p|+2 on the cell cycle was rescued in E2F1 ORF overexpressing cell lines (Fig. 6c). In summary, these results indicate that miR-183-5p|+2 directly targets – among others – the 3’UTR of *E2F1*, leading to the downregulation of E2F1, thereby causing reduced cell proliferation and repression of cell cycle progression. Of note, despite minor downregulation of E2F1 by miR-183-5p|+2, the impact on viability and cell cycle progression was comparable to the effects of *E2F1* knockdown indicating coordinate regulation of additional, yet unknown, targets cooperatively causing the phenotype of the isomiR.

### Pri-miR-183 transcription is regulated by E2F1

**Fig. 7:**
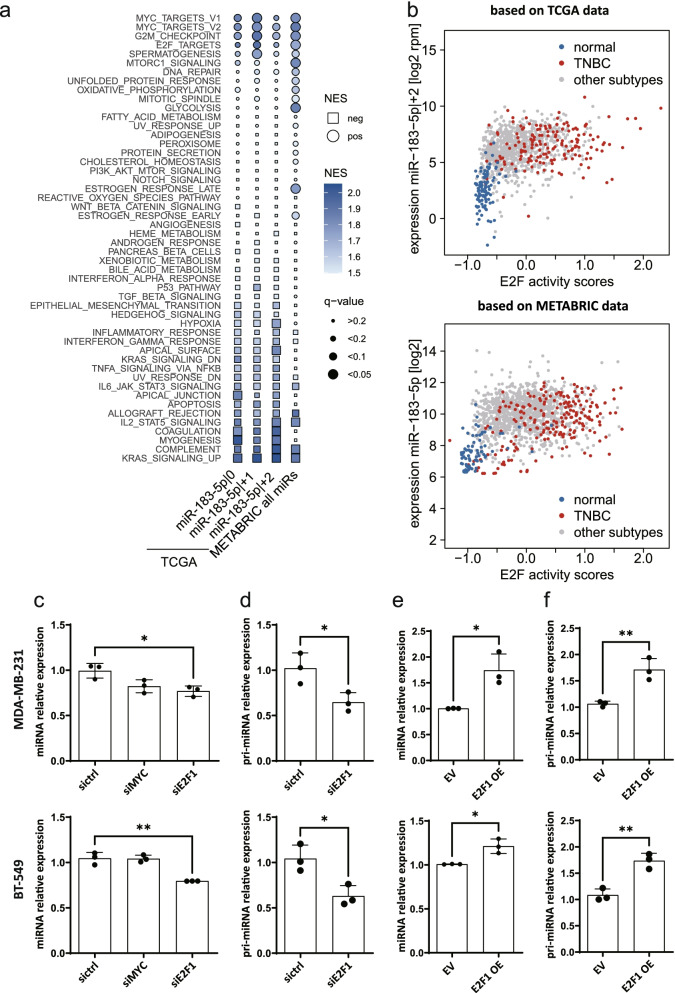
Transcriptional pri-miR-183 is regulated by the expression of *E2F1*. **a** Gene set enrichment analysis was performed on TCGA mRNA and miRNA sequencing data or METABRIC array-based expression data using the hallmark gene set collection and spearman correlation with the miRNA of interest as ranking metric. Normalized enrichment score (NES) and adjusted p-value of the respective enrichment analysis were visualized in a bubble heatmap. **b** An E2F activity score was computed for each patient based on the median z-scaled expression of all genes within the E2F target gene set. The relationship of E2F activity score and expression of miR-183-5p|+2 in TCGA or miR-183-5p in METABRIC data are depicted as scatterplots**.** Samples from normal tissue (blue) and from TNBC patients (red) were highlighted. **c-f** Downregulation of miR-183-5p and pri-miR-183 by knockdown of *E2F1* or overexpression of *E2F1*. MDA-MB-231 and BT-549 were seeded in 6-well plate for 72 h doxycycline-induction of E2F1 overepression or transfection (siE2F1 or siRNA negative control for 48 h) for knockdown. Total RNA was isolated using miRNeasy Kit (Qiagen). The mature miR-183-5p expression were quantified by miScript miRNA qPCR and normalized to *SNORD72* and *SNORD95*. Normalized expression is depicted as the relative expression to control **c**, **e**. The pri-miR-183 expression were quantified by SybrGreen-based RT-qPCR and normalized to housekeeping genes (*ACTB* and *PUM1*). Normalized gene expression is depicted as the relative expression to control **d**, **f**. Data are presented as mean ± SD, *n* = 3 (each derived from the median of 3 technical replicates). *p < 0.05, **p < 0.01 compared to control, unpaired *t-*test

Having observed direct targeting of *E2F1* by miR-183-5p|+2 in an *in vitro* setup, we hypothesized that the expression and activity of E2F1 should be inversely correlated with the expression of miR-183-5p|+2 in breast cancer patients. Therefore, we investigated the correlation of expression between miR-183-5p|+2 or miR-183-5p and *E2F1* mRNA levels in the TCGA and METABRIC datasets, respectively (Additional file 1: Supplementary Figure 7). However, the expression of *E2F1* itself did not exhibit a strong correlation with the expression of miR-183-5p|+2 (Spearman r = 0.19 in TCGA TNBC patients and r = 0.01 in METABRIC TNBC patients; Additional file 1: Supplementary Figure 7a). Since the post-transcriptional regulation via miRNAs may not affect the mRNA but only protein levels and thereby activity, we next assessed whether miR-183-5p|+2 expression would inversely correlate with the activity of E2F1 in the patient data. To this end, gene set enrichment analysis was applied to batch-corrected TCGA miRNA and mRNA sequencing data [33] for TNBC patients (Additional file 2: Supplementary Table 15 and Supplementary Table 16). In contrast to our expectations, E2F and MYC proto-oncogene, bHLH transcription factor (MYC) activities were positively correlated with the expression of all three isomiRs in TNBC patients in TCGA as well as with the overall expression of miR-183-5p in the METABRIC cohort (Fig. 7a, Additional file 1: Supplementary Fig. 7b). Of note, associated pathways differed substantially between PAM50 subtypes highlighting the importance of differentiated analysis of individual subtypes (Additional file 1, Supplementary Figure 8). Active E2F signaling was increased in tumor as compared to normal tissue and the expression of miR-183-5p|+2 was also higher in TNBC than in non-TNBC and normal tissue (Fig. 7b, Spearman correlation coefficient within TNBC patients r = 0.19 in TCGA and r = 0.36 in METABRIC; Additional file 2: Supplementary Table 14). The other two 5’isomiR-183-5p as well as miR-183-5p expression in the METABRIC cohort showed a similar trend (Fig. 7b, Additional file 1: Supplementary Figure 9a). Of note, the expression levels of the 5’isomiRs are highly correlated in patients suggesting that their expression is mainly regulated by transcriptional activity. Therefore, GSEA results, which are based on the correlations of genes with the respective isomiRs, are expected to be very similar for the three isomiRs and not indicative of similarities or dissimilarities between them. Similarly, activities of E2F and MYC are highly positively correlated in both TCGA and METABRIC breast cancer patient samples irrespective of their subtype (Spearman correlation coefficient r=0.82, both in TCGA and METABRIC datasets, Additional file 1: Supplementary Figure 9b). Hence, it cannot be inferred from this data if E2F, MYC or both are involved in the transcriptional regulation of pri-miR-183 – and thereby consistently of all three 5’isomiRs – in breast cancer patients.

To investigate this further, we quantified the levels of mature miR-183-5p upon knockdown of *E2F1* and *MYC.* Indeed, knockdown of *E2F1* led to reduced expression of both mature miR-183-5p and pri-miR-183 (Fig. 7c-d). In contrast, knockdown of *MYC* did not significantly affect the expression of mature miR-183-5p indicating that MYC is not the relevant transcription factor in our model systems. Accordingly, the expression of miR-183-5p and of pri-miR-183 was increased upon overexpression of *E2F1* (Fig. 7e-f). Together, these results point towards a regulatory circuit comprising of transcriptional upregulation of miR-183 which in turn can lead to post-transcriptional downregulation of E2F1.

### Discussion

MicroRNAs, as post-transcriptional regulators, influence tumor behavior and progression [16]. High throughput sequencing has demonstrated the existence of isomiRs, which are length and/or sequence variants of miRNAs. Of note, 5’isomiRs exhibit a shifted seed sequence compared to their canonical counterparts leading to an altered target spectrum, which has been rarely taken into consideration in previous research [14]. The functional relevance of 5’isomiRs might thus be underestimated, but should be considered towards a more holistic understanding of the mechanisms underlying tumor progression. Sequencing data from the TCGA breast cancer cohort indicated that three variants of miR-183-5p are highly upregulated in breast cancer compared to normal breast tissue, namely miR-183-5p|0, miR-183-5p|+1 and miR-183-5p|+2. Due to the substantial intrinsic differences between breast cancer subtypes, we focus here specifically on TNBC for mechanistic studies. Patient data analysis indicated that phenotypic traits of tumor cells associated with expression levels of miR-183 strongly differ between the PAM50 subtypes of breast cancer (Additional file 1: Supplementary Figure 8). Yet, this does not imply that other subtypes are not similarly affected by expression of miR-183-5p.

*In vitro*, overexpression of pre-miR-183 in both MDA-MB-231 and BT-549 cell lines reduced cell migration and invasion. These results are in line with a previous study on the tumor-suppressive role of miR-183-5p in lung cancer, where *Wang et al.* found that overexpression of miR-183 inhibited migration and invasion by targeting *EZR* [45]. Another study in HeLa cells indicated that overexpression of miR-183 caused a significant decrease in cell migration and invasion [46]. In contrast, several other studies have claimed that miR-183, functioning as an oncogene, contributes to the tumorigenesis of various cancers. For instance, *Sarver et al*. showed that downregulation of miR-183 suppressed *EGR1* and *PTEN* in synovial sarcoma, rhabdomyosarcoma, and colon cancer cell lines, and was accompanied by decreased cell migration and invasion in both systems [47]. Besides, in gastric cancer cells, miR-183 increased cell proliferation and decreased cell autophagy and apoptosis by regulating *UVRAG* [48]. Taken together, this controversy emphasizes the context-dependent function of miRNAs as their functions depend on both expression levels and the importance of their targets in the cell. Importantly, previous studies on miR-183 in breast cancer and other tumor entities did not consider 5’isomiRs [49, 50], which adds another layer of complexity and regulatory potential. In our study, overexpression of each individual 5’isomiRs of miR-183-5p inhibited cell migration and invasion to similar extents. However, overexpression of pre-miR-183 in an *in vivo* xenograft model did not alter the potential of MDA-MB-231 cells to metastasize to the lung in our experimental setup. Yet, we observed a trend of reduced tumor growth that failed to reach statistical significance with the limited number of mice used.

*In vitro,* we observed that overexpression specifically of miR-183-5p|+2 led to a decrease in cell viability and impaired ability of cells to re-enter cell cycle employing three genetically diverse models of TNBC, emphasizing the generality of this finding. Especially in the BT-549 cell line, not only miR-183-5p|+2 but also the other two 5’isomiRs of miR-183-5p inhibited cell proliferation. This might be explained by the genetic and phenotypic heterogeneity of the model cell lines. Potentially, BT-549 expresses a different set of target genes or is addicted to a specific pathway regulated by miR-183-5p|0 and |+1. Taken together, among the miR-183-5p 5’isomiRs, miR-183-5p|+2 displays the strongest tumor-suppressive effect in cell viability and cell cycle. These results are in line with our hypothesis that 5’isomiRs with a shifted seed sequence have different target spectra potentially resulting in a functional difference compared to their canonical counterparts as well as to other 5’isomiRs. This is in line with a study of *Telonis et al.* who surveyed transcriptome-differences of three different 5’isomiR-183-5p in MDA-MB-231 [51]. Overexpression of distinct miR-183-5p isomiRs in MDA-MB-231 cells followed by microarray analysis revealed that each isomiRs of miR-183-5p had a different targetome and the effect of the isomiRs on the transcriptome differed drastically. However, these authors restricted their investigation to computational analyses and did not functionally validate their findings [51]. In our present study, we have extended this analysis to the proteome of MDA-MB-231 cells. In addition, we have proven that 5’isomiR-183-5p exhibit functional differences in cell viability and cell cycle of TNBC. This is well in line with a growing number of reports pointing out that 5’isomiRs exhibit differential functions. A previous study in our group showed that 5’isomiR-140-3p specifically inhibits proliferation and migration by directly regulating targets distinct from the canonical miRNA [15]**.** Another study conducted by *Bhardwaj et al.* showed that miR-140-3p|+1 directly regulates *HMGCR* and *HMGCS1* leading to inhibition of cell growth [52] thus confirming that 5’isomiRs frequently have different functions than canonical miRNAs.

Since it is widely accepted that the functions of miRNAs rely on their target genes, we hypothesized that the phenotypic differences were due to distinct target genes. Commonly, potential targets of miRNA are predicted based on sequence complementarity using computational tools, such as miRanda, PicTar and TargetScan. However, these prediction tools frequently identify a vast amount of false-positive candidates [53] and predictions for miR-183-5p|+2 were not available from these databases. To overcome shortcomings of computational methods, we performed custom target predictions combining consensus predictions from miRanda and TargetScan restricted to bona-fide expressed 3’UTRs. Since the regulation of target genes by a miRNA ultimately leads to changes in protein expression, we chose mass spectrometry analysis to identify direct and indirect targets of miR-183-5p in this study. Mass spectrometry analysis showed that overexpression of miR-183-5p|+2 resulted in protein expression patterns distinct from the other two 5’isomiR-183-5p and also from the controls. While this analysis cannot discriminate between direct and indirect miRNA-effects, it clearly indicates a global functional difference between the isoforms. Here, we demonstrate the post-transcriptional regulatory effects of the isomiRs on protein level. Yet, we cannot make claims about their impact on global target mRNA stability due to lack of transcriptomics data. Gene set enrichment analysis based on mass spectrometry data suggested that the E2F target signature was specifically decreased by the overexpression of miR-183-5p|+2, indicating a potential molecular mechanism underlining the functional difference among the three investigated 5’isomiR-183-5p. Of note, we also observed a highly significant downregulation of proteins belonging to the hallmark gene set ‘G2/M Checkpoint’ by miR-183-5p|+2 suggesting a second, yet unexplored, impact of miR-183-5p|+2 on cell cycle progression. Similarly, the ‘Hypoxia’ gene set appeared to be positively associated with overexpression of this 5’isomiR based on our proteomic data. However, these observations were out of the scope of the current study and require further validation to confirm their relevance. It is important to note that we here employed gene sets derived from transcriptomic signatures and transcription factor binding site predictions together with proteomic data. Therefore, associations observed in this analysis require additional validation as provided in this study in case of the regulation of E2F by miR-183-5p.

E2Fs, a group of genes that encode a family of transcription factors, are vital for cell cycle regulation, especially for the transition from G0/G1- to S-phase [54]. Based on the stringent target prediction, we found that *E2F1* is a predicted specific target of miR-183-5p|+2 and confirmed this by a 3’UTR luciferase reporter assay. Besides, we showed that siRNA-mediated knockdown of *E2F1* partially phenocopied the effect of miR-183-5p|+2 on cell proliferation and cell cycle re-entry. This is congruent with a study demonstrating that upregulation of *E2F1* promoted G1/S transition and induced tumorigenesis of TNBC [55]. Taken together, these results indicate a tumor-suppressive function of miR-183-5p|+2 by directly targeting *E2F1*. On the other hand, these results also highlight that miRNA effects cannot be explained by individual direct targets but rather by coordinate regulation of multiple proteins contributing to the same phenotype. This concept has been demonstrated for several example miRNAs [56, 57]. Therefore, it is plausible that also in case of miR-183-5p, various downregulated targets contribute to the observed phenotypes. Yet, it is out of scope of the present study to identify and validate these additional targets.

To investigate the potential clinical relevance of miR-183-5p|+2 targeting *E2F1*, gene set enrichment analysis was used to determine the correlation of E2F1 activity and miR-183-5p in TCGA and METABRIC TNBC patients’ data. These analyses showed that E2F and MYC activities positively correlated with the expression of all three 5’isomiR-183-5p, which contrasts our expectation to observe an inverse correlation of *E2F1* and miR-183-5p|+2. Therefore, we hypothesized that expression of miR-183 might, in turn, be regulated by either E2F or MYC resulting in the observed positive correlation in patients. Along this line, we validated that the expression of mature miR-183 and its precursor pri-miR-183 was indeed regulated by E2F1. This is in line with recent miRNA sequencing data demonstrating a 60% decrease in expression of miR-183-5p upon knockdown of *E2F1* in HeLa cells [58]. Of note, the oncogenic miRNAs miR-182 and miR-96 originating from the same primary transcript as miR-183 are coordinately downregulated by knockdown of *E2F1* in this dataset. This further supports transcriptional regulation of these miRNAs by E2F1. Together, we demonstrated both direct targeting of *E2F1* by miR-183-5p|+2, and transcriptional regulation of miR-183 by E2F1 *in vitro* in TNBC cell lines, and patient data analysis supports the upregulation of miR-183 in tumors with high E2F activity. This strongly indicates the presence of a negative feedback loop between these two components. Yet, the analysis of steady state expression data of breast cancer patients does not enable a more precise characterization of this feedback loop in patient tumors. In addition, the exact characteristics of the regulation likely differ between individual patients based on their mutational backgrounds (e.g. in the E2F1 regulating *RB1* gene). This and if additional targets of miR-183 are involved in the feedback regulation cannot be concluded here and would require additional research.

In our system, MYC seemed not to significantly influence the expression of miR-183. This does not exclude the possibility that MYC might be a regulator of miR-183 expression in other cellular contexts as well as in patients. Given its oncogenic roles in tumor progression, it is not surprising that E2F1 activity is higher in breast cancer tissue as compared to normal tissue with the highest activity in aggressive TNBC tumors. Consequently, this results in the increased expression of miR-183 which might not be expected given its rather tumor-suppressive role in different systems [45, 46]. Interestingly, we could demonstrate that miR-183-5p|+2 directly targets *E2F1* and leads to downregulation of E2F1 expression within a negative feedback loop, thereby potentially restricting cell proliferation by repressing cell cycle progression. This might be especially relevant in cells deficient in pRB protein. Under physiological conditions, this protein is crucial in the control of cell cycle entry by sequestering E2F transcription factors. In cancer, especially in TNBC, the expression and functionality of pRB are frequently disrupted by various mechanisms [59]. Hence, these cells can enter cell cycle independent from activating signals. Here, it could be speculated that the feedback regulation by miR-183-5p|+2 might be part of an evolutionary fail-safe mechanism to initially prevent overactive E2F, potentially also during extensive mitogenic signalling.

Our study highlights that due to the heterogeneity and complexity of tumorigenesis, the high expression of a miRNA in tumors is not necessarily connected to an oncogenic function of this miRNA. Such elevated expression might rather be an indication of a feedback loop that is supposed to counteract the growth-promoting activity of the miRNA-targets under physiological conditions. Given the success of tumor cells in overcoming negative regulation of growth, the observed elevated expression of miR-183-5p could be seen as a reminiscence of a negative control mechanism that has obviously failed in the progressed tumor system. Concretely, the suppression of E2F1 by miR-183-5p|+2 could potentially be compensated by upregulation of other E2F transcription factors or loss of pRB, which might in turn further upregulate expression of miR-183.

## Conclusion

In summary, we demonstrated that miR-183-5p is highly upregulated in aggressive triple-negative breast cancer and that high expression is associated with tumor-suppressive traits of TNBC cells *in vitro*. Specifically, we could link direct targeting of *E2F1* by miR-183-5p|+2 to reduced proliferation, thereby establishing a dynamic negative feedback loop in TNBC where E2F1 in turn upregulates expression of miR-183-5p. These findings highlight the complex nature of miRNA-target interactions and their downstream effects. These aspects require further biological validation to approach a comprehensive understanding of the role of miRNAs in cancer progression.

## Supplementary Information

**Additional file 1 : Supplementary Fig. 1.** Tumor metastasis and growth *in vivo*. **Supplementary Fig. 2.** Cell death in TNBC cells upon isomiR overexpression. **Supplementary Fig. 3.** Gene set enrichment analysis of mass spectrometry data. **Supplementary Fig. 4.** Schematic representation of the predicted target site for miR-183-5p|+2 in the 3’UTR of E2F1. **Supplementary Fig. 5.** Knockdown efficiency of siE2F1 on the protein level. **Supplementary Fig. 6.** Validation of *E2F1* overexpressing TNBC cell lines by Western Blot. **Supplementary Fig. 7.** Correlation plots of *E2F1* and *MYC* expression and GSEA plots for the correlation of E2F targets and MYC targets gene sets with miR-183-5p in TCGA and METABRIC datasets. **Supplementary Fig. 8.** Bubble Heatmap of GSEA results of gene sets associated with miR-183-5p expression in different PAM50 subtypes in TCGA and METABRIC. **Supplementary Fig. 9.** Correlation of E2F and MYC activity scores with miR-183-5p in patient datasets.

**Additional file 2 : Supplementary Table 1.** miRNA mimics and siRNAs. **Supplementary Table 2.** Cloning Primers. **Supplementary Table 3.** qPCR Primers and Probes. **Supplementary Table 4.** Antibodies. **Supplementary Table 5.** Luciferase Assay Buffers. **Supplementary Table 6.** isomiR Differential Expression Analysis. **Supplementary Table 7.** pre-miR-183 overexpression levels in stable cell lines. **Supplementary Table 8.** Statistical Analysis of Fig. 3d-f. **Supplementary Table 9.** MassSpec Raw Data (LFQ, non-imputed). **Supplementary Table 10.** MassSpec ranked expression files used for GSEA. **Supplementary Table 11.** GSEA results comparing protein expression between isomiRs and controls and using the MSigDB Hallmark gene set collection. **Supplementary Table 12.** Z-scores of LFQ intensities for the E2F1 targets (heatmap, Fig. 4c). **Supplementary Table 13.** Results of Fisher's exact test. **Supplementary Table 14.** Target Prediction results. **Supplementary Table 15.** TCGA-BRCA and METABRIC mRNA and miRNA expression data and activation scores for selected genes and miRs. **Supplementary Table 16.** GSEA results comparing gene expression from TCGA and METABRIC patient data based on expression of isomiRs of miR-183-5p of miR-183-5p in METABRIC.

**Additional file 3 .** Detailed Methods.

## Data Availability

Data presented in the manuscript including Target Predictions, batch-corrected isomiR expression, E2F activity scores in TCGA-BRCA patients and a summary of the GSEA analyses are collected in Additional file 2. Data obtained from Mass Spectrometry have been deposited to the ProteomeXchange Consortium [60] via the PRIDE [61, 62] partner repository with the dataset identifier PXD026763. Overexpression constructs can be obtained from the authors.

## Abbreviations

miRNA: MicroRNA
TCGA: The Cancer Genome Atlas
TNBC: Triple-negative breast cancer
E2F1: E2F transcription factor 1
MYC: MYC proto-oncogene, bHLH transcription factor
Pri-miRNA: Primary microRNA
Pre-miRNA: Precursor miRNA
UTR: Untranslated region
FBS: Fetal bovine serum
NEAA: Non-Essential Amino Acid
NSG: NOD-SCID IL-2 receptor gamma null
BrdU: Bromodeoxyuridine
7-AAD: 7-Aminoactinomycin D
LFQ: Label-free quantification
qRT-PCR: Quantitative real time polymerase chain reaction
GSEA: Gene set enrichment analysis
NES: Normalized enrichment score
SD: Standard deviation
