# Supplementary material for: 5’isomiR-183-5p|+2 elicits tumor suppressor activity in a negative feedback loop with E2F1
Source: J Exp Clin Cancer Res. 2022 Jun 2;41:190. doi: 10.1186/s13046-022-02380-8 (PMC9161486; doi:10.1186/s13046-022-02380-8)
Supplement: Supplementary file 1 — Additional file 1 : Supplementary Fig. 1. Tumor metastasis and growth in vivo. Supplementary Fig. 2. Cell death in TNBC cells upon isomiR overexpression. Supplementary Fig. 3. Gene set enrichment analysis of mass spectrometry data. Supplementary Fig. 4. Schematic representation of the predicted target site for miR-183-5p|+2 in the 3’UTR of E2F1. Supplementary Fig. 5. Knockdown efficiency of siE2F1 on the protein level. Supplementary Fig. 6. Validation of E2F1 overexpressing TNBC cell lines by Western Blot. Supplementary Fig. 7. Correlation plots of E2F1 and MYC expression and GSEA plots for the correlation of E2F targets and MYC targets gene sets with miR-183-5p in TCGA and METABRIC datasets. Supplementary Fig. 8. Bubble Heatmap of GSEA results of gene sets associated with miR-183-5p expression in different PAM50 subtypes in TCGA and METABRIC. Supplementary Fig. 9. Correlation of E2F and MYC activity scores with miR-183-5p in patient datasets. [file 13046_2022_2380_MOESM1_ESM.pdf]

Additional File 1: Supplementary Figures

Supplementary Figure 1

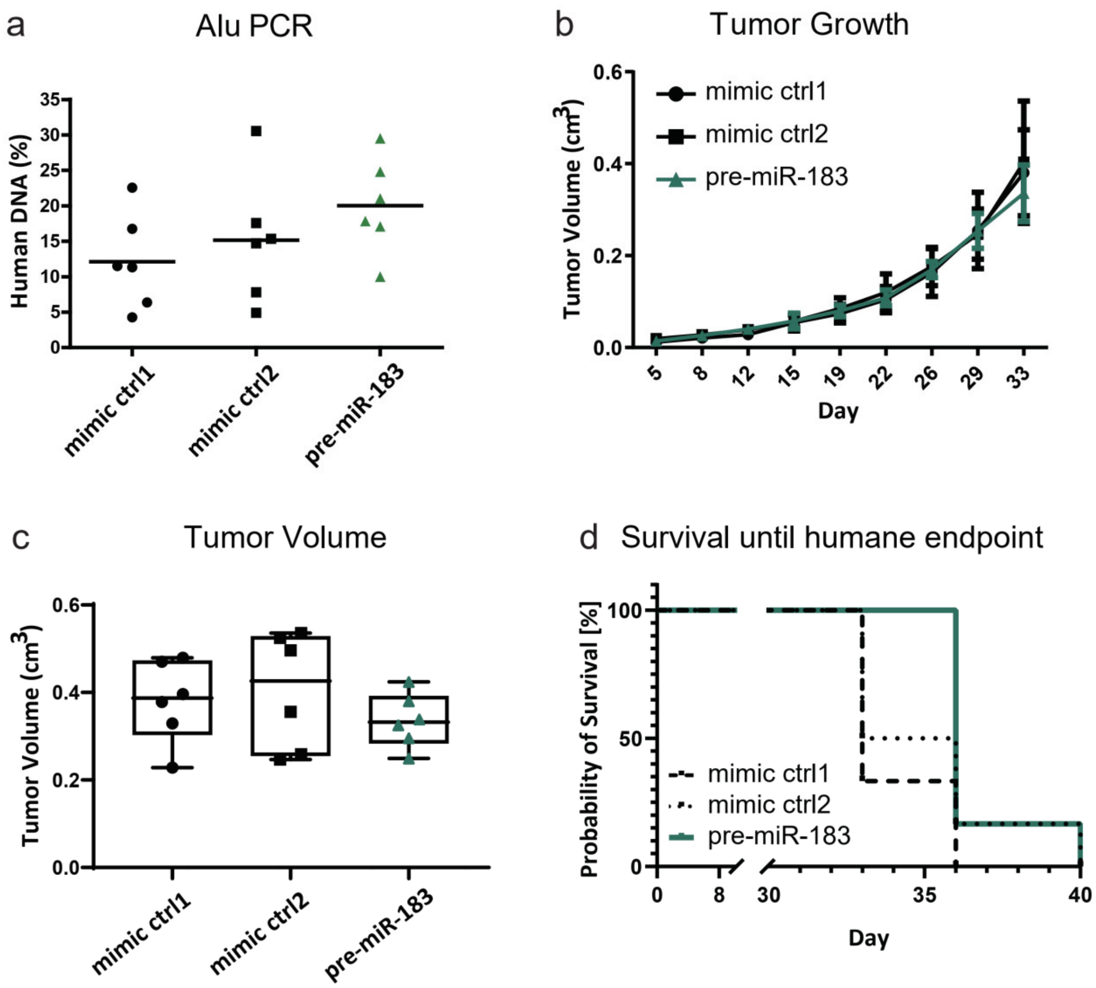

Supplementary Figure 1: Tumor metastasis and growth *in vivo*. MDA-MB-231 cells stably overexpressing control miRNAs or pre-miR-183 were orthotopically injected into the mammary fat pad of NSC mice. (a) Mice were sacrificed when reaching the humane endpoint and DNA from lungs was isolated to quantify metastases by Alu PCR. (b) Tumor growth was monitored twice a week starting at the time of doxycycline induction (33 days post doxycycline induction). (c) Tumor volume at the time when the first tumors reached the humane endpoint of 1cm. The trend of reduced tumor growth with overexpression of miR-183 was not significant. (d) Survival in days after doxycycline induction until the humane endpoint (pre-miR-183 vs ctrl1: $p<0.05$; pre-miR-183 vs ctrl2: not significant).

Supplementary Figure 2

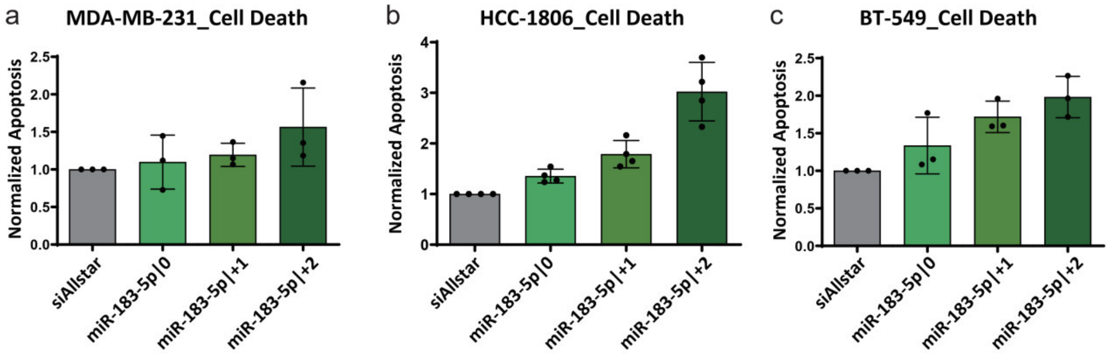

Supplementary Figure 2: Cell death. (a) MDA-MB-231, (b) HCC-1806 and (c) BT-549 cells were seeded into clear-bottom 96-well black plates and transfected with miRNA mimics or siAllstar. 72h after transfection, the cells were stained with Hoechst 33342 and propidium iodide and quantified using Molecular Devices Microscope IXM XLS. The cell number of viable cells was measured by counting cell nuclei based on Hoechst signal. Cells positive for propidium iodide staining were defined as apoptotic cells. Values were normalized to the respective control. Data are presented as mean ± SD, n = 3 (each derived from the median of 6 technical replicates). Statistical analysis: ordinary one-way ANOVA followed by Dunnett's multiple comparisons test $^{*}p < 0.05$, $^{**}p < 0.01$, $^{***}p < 0.001$ compared to control.

Supplementary Figure 3

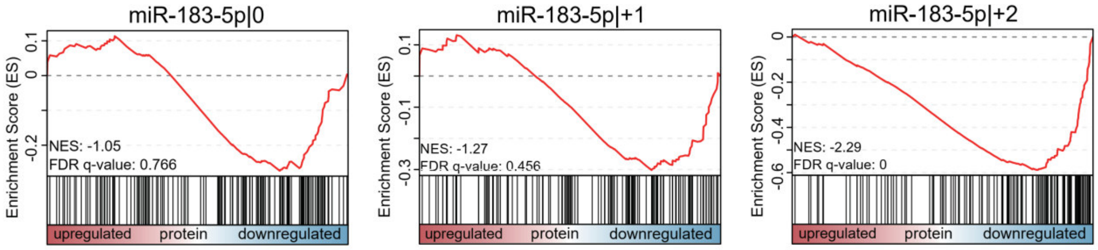

Supplementary Figure 3: Gene set enrichment analysis of mass spectrometry data. Mass spectrometry derived data from MDA-MB-231 cells overexpressing one of the 5'isomiRs of miR-183-5p were used as input for Gene Set Enrichment Analysis using preranked GSEA and log2 foldchange as ranking metric.

Supplementary Figure 4

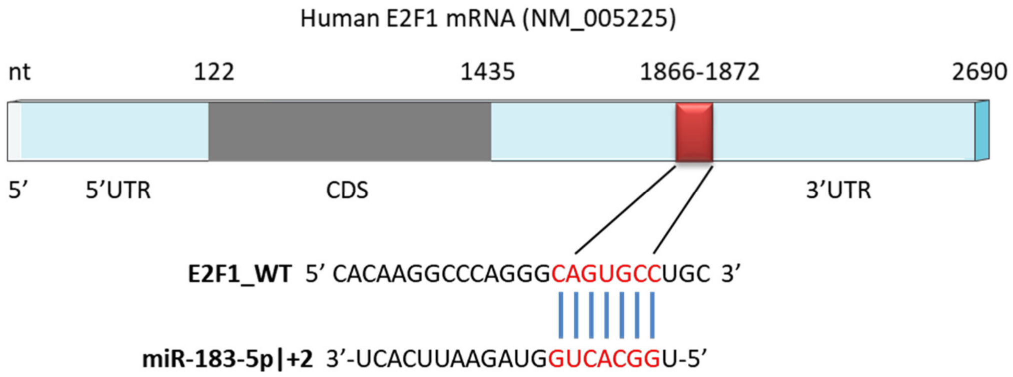

Supplementary Figure 4: Schematic representation of the predicted target site for miR-185-5p|+2 in the 3'UTR of *E2F1*.

Supplementary Figure 5

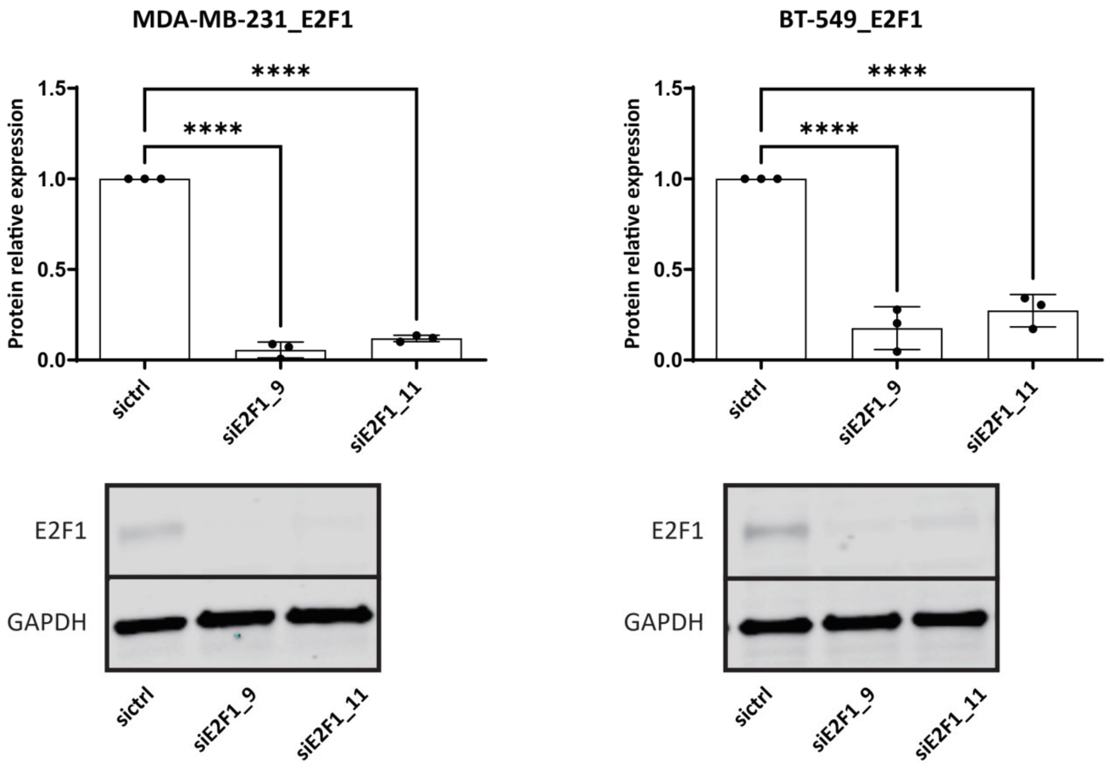

Supplementary Figure 5: Knockdown efficiency of siE2F1 on the protein level. MDA-MB-231 and BT-549 cells were transfected with two individual siRNAs targeting E2F1 and corresponding protein lysates were subjected to Western Blot analysis using the LiCor Odyssey system. Statistical analysis: ordinary one-way ANOVA followed by Dunnett's multiple comparisons test, ****p < 0.0001.

Supplementary Figure 6

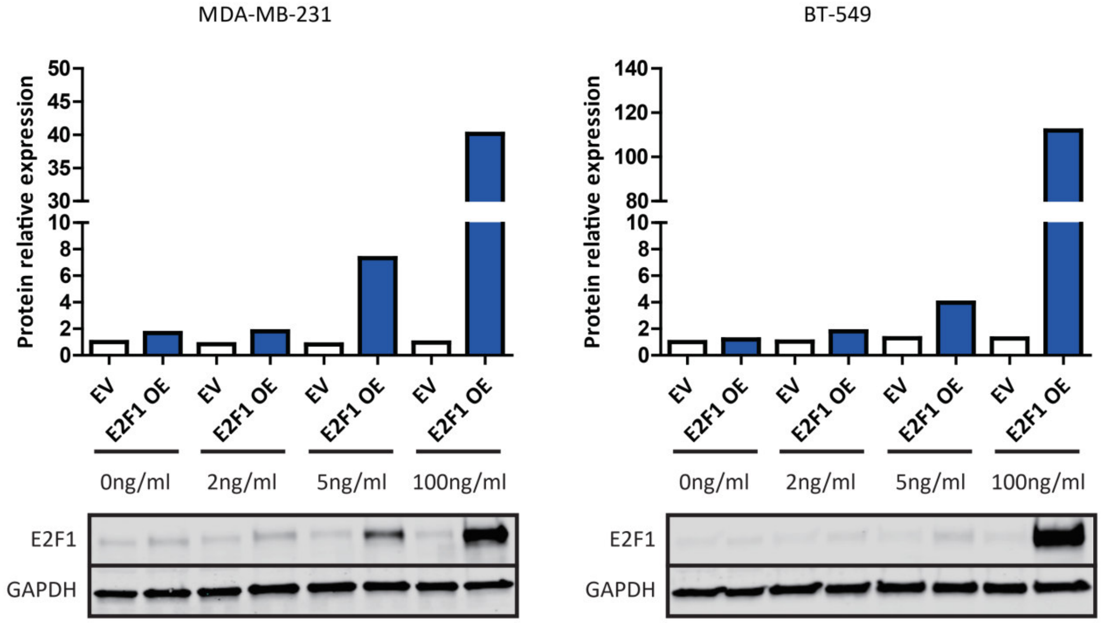

Supplementary Figure 6: Validation of E2F1 overexpressing TNBC cell lines by Western Blot. MDA-MB-231 and BT-549 stably transduced with doxycycline-inducible *E2F1* were induced for 48h using the indicated concentrations of doxycycline. Protein lysates were subjected to Western Blot analysis using the LiCor Odyssey system. EV: empty vector control, OE: overexpression.

Supplementary Figure 7

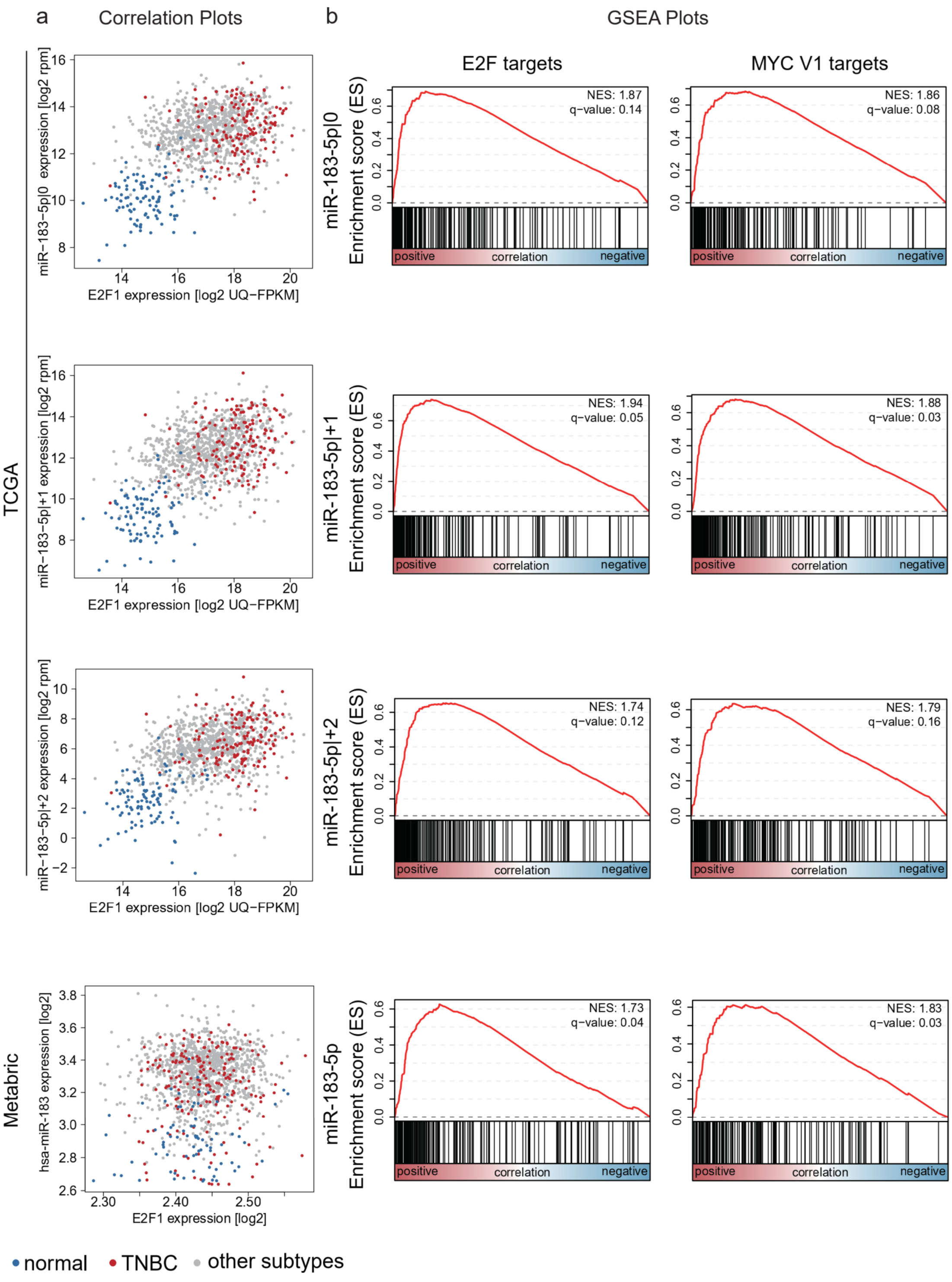

Supplementary Figure 7: Association between *E2F1* and miR-183 in breast cancer patients. (a) Correlation plots for E2F1 expression and miR-183-5p in TCGA and METABRIC datasets. TNBC patients are displayed in red, tumor-adjacent normal tissue samples are displayed in blue. All other tumor samples are shown in grey. (b) GSEA plots for the correlation of E2F targets and MYC V1 targets gene sets with miR-183-5p in TCGA and METABRIC datasets. Gene set enrichment analysis was performed on TCGA mRNA and miRNA sequencing data or METABRIC array-based expression data using the hallmark gene set collection and Spearman correlation with the miRNA of interest as ranking metric. Corresponding GSEA plots for the E2F target and the MYC V1 targets are shown.

Supplementary Figure 8

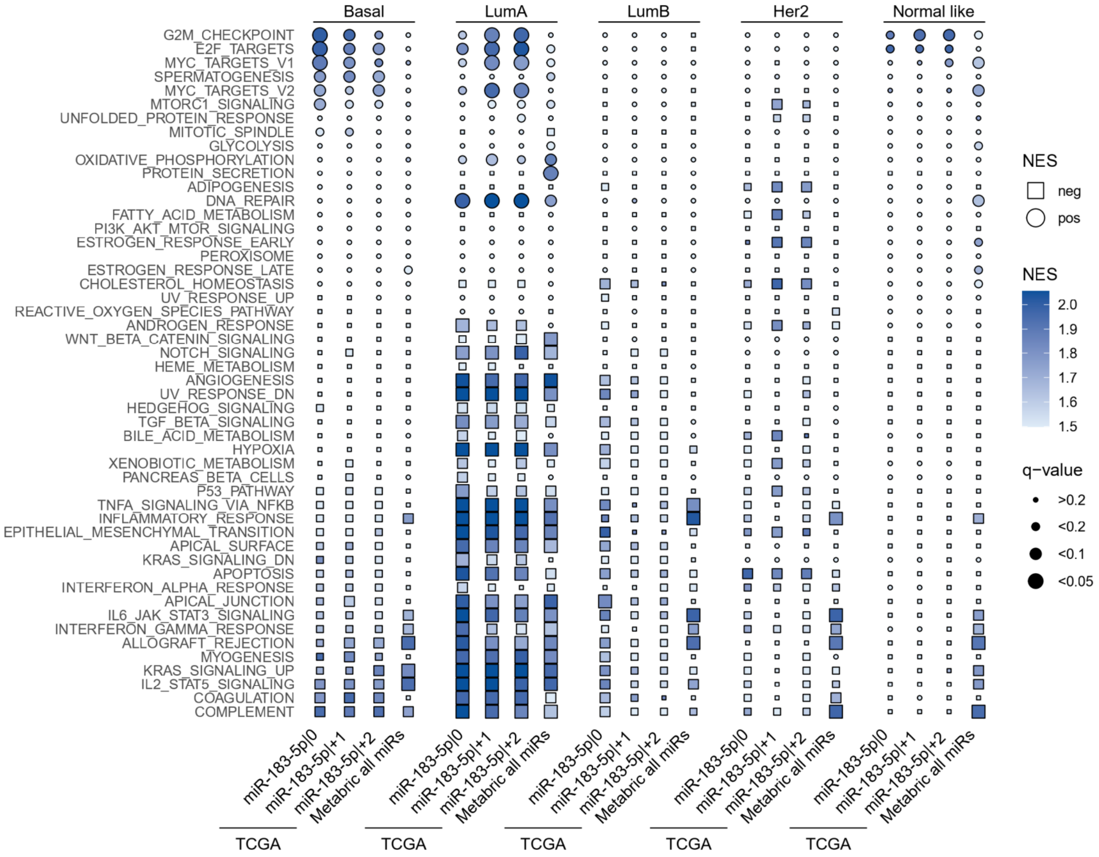

Supplementary Figure 8: Bubble Heatmap of GSEA results of gene sets correlated with miR-183-5p expression in different PAM50 subtypes in TCGA and METABRIC. GSEA was performed within individual PAM50 subtypes of breast cancer for the Hallmark gene set collection of the Molecular Signatures Database as detailed in Materials and Methods. Briefly, Spearman correlation between genes and the indicated isomiR of miR-183-5p (TCGA) or the probe for miR-183-5p (METABRIC) was used as ranking metric. Statistical analysis was performed by phenotype permutation. Normalized Enrichment Score (NES) and FDR q-values were summarized as bubble heatmap.

Supplementary Figure 9

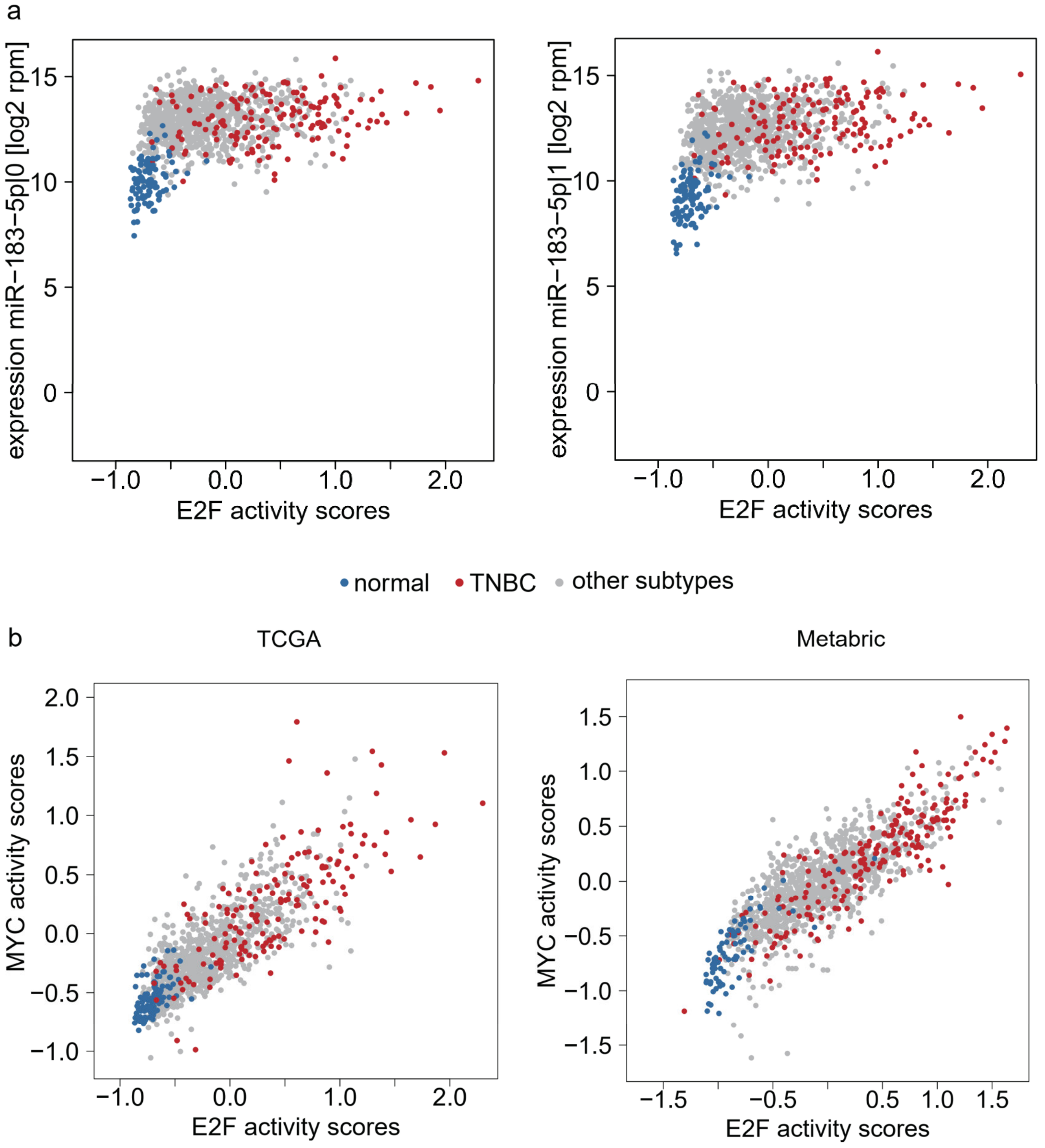

Supplementary Figure 9: (a) Association of E2F activity scores with expression of miR-183-5p|0 and |1 in TCGA patients' data. An E2F activity score was computed for each patient based on the median z-scaled expression of all genes within the E2F target gene set. The relationship of E2F activity score and expression of miR-183-5p|0 and |+1 in TCGA data are depicted as scatterplots. Samples from normal tissue (blue) and from TNBC patients (red) were highlighted. **(b) Association of E2F activity scores with MYC activity scores in TCGA and METABRIC datasets.** A MYC activity score was computed for each patient based on the median z-scaled expression of all genes within the MYC target gene set (v1). The relationship of E2F and MYC activity scores in TCGA and METABRIC data are depicted as scatterplots. Samples from normal tissue (blue) and from TNBC patients (red) were highlighted.
