# Supplementary material for: 5’isomiR-183-5p|+2 elicits tumor suppressor activity in a negative feedback loop with E2F1
Source: J Exp Clin Cancer Res. 2022 Jun 2;41:190. doi: 10.1186/s13046-022-02380-8 (PMC9161486; doi:10.1186/s13046-022-02380-8)
Supplement: Supplementary file 3 — Additional file 3 . Detailed Methods. [file 13046_2022_2380_MOESM3_ESM.pdf]

Additional file 3 - Detailed Methods

Transfection

Cells were seeded at 70-80% confluency one day before transfection. All transfections (plasmids, siRNAs or miRNA mimics) were performed using Lipofectamine2000 (LF2000) according to the manufacturer's instructions (0.4 µL/well in 96-well plates, 4 µL/well in 6well plates and 10 µL/dish in 10 cm dishes). The transfection procedure is first to mix the appropriate amount of LF2000 with OptiMEM. The LF2000-OptiMEM pre-mix was incubated at room temperature for 15 min. During this incubation time, plasmids or RNAs were diluted in OptiMEM to a final volume equal to pre-mix and added to LF2000-OptiMEM pre-mix for 20 min at room temperature. Meanwhile, medium was aspirated from the cells and replaced by OptiMEM. The transfection mix was added dropwise to the cells. After incubation for 5 h at 37°C, 5% CO2, the transfection medium was replaced by fresh full growth medium. Cells were incubated for 48 or 72 h at 37 °C, 5% CO2 in a humidified atmosphere before they were used for experiments.

All siRNAs (Dharmacon, Lafayette, USA) and synthesized miRNA mimics (Qiagen, Hilden, Germany) were used at a final concentration of 30 nM. The siRNAs purchased from siTOOLs Biotech (Planegg, Germany) were used at a final concentration of 2 nM. The sequences of siRNAs and miRNA mimics are listed in Supplementary Table 1.

Generation of stable cell lines

To generate TNBC cell lines overexpressing pre-miRNAs (pre-miR-183 or two different pre-miRNA negative controls), the pre-miRNAs were cloned into a retroviral vector RT3GEPIR [1]. The sequences of primers for cloning are listed in Additional file 2: Table 2. Retroviral particles were produced in HEK293FT cells by co-transfection of the retroviral RT3GEPIR vector, together with the VSV.G envelope plasmid pMD2.G (Addgene #12259) and gag/pol packaging plasmid pHIT60 [2].

To generate inducible E2F1 overexpressing TNBC cell lines, the open reading frame of human *E2F1* (Addgene plasmid # 70329) was shuttled into the lentiviral expression vector rwSMART-TRE3G-GW-mCMV-TetON3G (Cellular Tools DKFZ, Heidelberg, Germany) using Gateway cloning technology [3] (ThermoFisher, Braunschweig, Germany). E2F1 expression is controlled by a doxycycline inducible promoter (TRE3G) which allows a tight regulated and concentration dependent E2F1 expression in the presence or absence of doxycycline. Lentiviral particles were produced in HEK293FT cells by co-transfection of the lentiviral rwSMART-TRE3G-E2F1-mCMV-TetON3G expression vector, together with 2nd generation viral packaging plasmids VSV.G (Addgene #14888) and psPAX2 (Addgene #12260).

24 h after transfection, the medium of HEK293FT was replaced by full growth medium of the target cell line. After 24 h, virus-containing supernatant was collected and centrifuged at 500 g for 5 min. The supernatant was filtered with a 0.45 µm filter to remove remaining cellular debris. Target cells were transduced with filtered supernatant in the presence of 10 µg/mL polybrene (Merck, Germany). 24 h after

transduction, virus-containing medium was replaced with selection medium (full growth medium containing 2 µg/mL puromycin).

Transwell-based cell migration and invasion assay

After pre-treatment (miRNA mimic and doxycycline-induction), cells were starved in 0% FBS starvation medium for 24 h. Then, 100,000 cells in 200 µL of 0% FBS starvation medium were seeded into the upper compartment of Transwell inserts (Corning, Kaiserslautern, Germany). Specifically, for cell migration assay, the Transwell inserts with 5.0 µm or 8.0 µm pore sizes were used for MDA-MB-231 or BT-549, respectively. For cell invasion assay, the Transwell inserts with 8.0 µm pore size which were pre-coated with 5 mg/mL matrigel were used for MDA-MB-231 and BT-549. 500 µL of full growth medium was used in the lower compartment as chemoattractant. Cells were allowed to migrate or invade for 16 h. In parallel, 1:50 dilutions of the seeding cells were seeded into black clear-bottom 96-well plates as a seeding control plate for normalization. The cells on the lower side of the membrane were fixed with 4% PFA (16% Formaldehyde (w/v), 1:4 dilution in PBS) for 15 min and stained with Hoechst 33342 (4 µM in growth medium) for 30 min. The seeding control plate was also stained with Hoechst 33342 in the same dilution. Subsequently, the seeding control plate and the inserts were imaged with Molecular Devices Microscope IXM XLS (Molecular Device, California, USA) using 4x S Fluor objective. The cell number was obtained by counting cell nuclei on each image. All nuclei were defined by Hoechst signals within a certain size (6-35 µm) and intensity (5000 gray levels above local background) and counted using Molecular Devices Software (Molecular Device, California, USA). Afterwards, the exemplary membranes were stained with 0.5% crystal violet for 30 min and then imaged with microscope.

Cell proliferation assay

Cell proliferation was analyzed with a microscope-based nuclei counting method. Cells were pre-treated with miRNA mimic, siRNA or doxycycline-induction. After pre-treatment, cells were starved in 0% FBS starvation medium for 24 h. Starved cells were harvested and 1000 cells were seeded with full growth medium into black clear-bottom 96-well plates. At different time points, cell nuclei were stained with Hoechst 33342 (20 mM in stock, 1:5000 dilution in growth medium) for 30 min. Subsequently, the plates were imaged with Molecular Devices Microscope IXM XLS (Molecular Device, California, USA) using 4x S Fluor objective. The cell number was obtained by counting cell nuclei on each image. All nuclei were defined by Hoechst signals within a certain size (6-35 µm) and intensity (5000 gray levels above local background) and counted with Molecular Devices Software (Molecular Device, California, USA).

BrdU/7ADD based cell cycle assay

Cell cycle phases were analyzed with Bromodeoxyuridine (BrdU) to stain cells in S phase and 7-Aminoactinomycin D (7-AAD) to stain the whole cellular DNA content. Cells were pre-treated (miRNA mimic, siRNA and doxycycline-induction). After pre-treatment, cells were starved in 0% FBS starvation medium for 24 h to synchronize the cell cycle distribution. After starvation, the cells were released from cell cycle block with

full growth medium for 24 h and incubated with 10 µM BrdU (10 mg/mL, 32.5 mM in stock) 2 h prior to harvest. After BrdU incorporation, the cells were permeabilized by the Perm/Wash buffer and fixed with 250 µL Cytofix/Cytoperm buffer for 20 min at room temperature. Afterwards, 150 µL DNase (300 µg/mL) was added and incubated for 1 h at 37°C to expose the BrdU. Anti-BrdU was added and incubated for 20 min at room temperature for detection (10 µL BrdU-FITC mixed with 50 µL PBS was used for parental cells. 5 µL BrdU-APC mixed with 55 µL PBS was used for GFP-expressing stable cell lines). Following detection, cells were incubated with 7-AAD for 30 min at room temperature in the dark. Samples were subjected to flow cytometric analysis using CellQuest Pro (BD Biosciences, USA) and BD FACS DIVA software (BD Biosciences, USA).

RNA extraction and qRT-PCR

RNA was isolated using the RNeasy or miRNeasy Kit (Qiagen, Hilden, Germany) according to the manufacturer's recommendations. The concentration of total RNA was determined by NanoDrop ND-1000.

To prepare cDNA for subsequent TaqMan or SybrGreen-based qPCR, the RevertAidTM H minus First-strand Kit (Thermo Fischer Scientific, Massachusetts, USA) was used. For TaqMan assay, primers and probes were designed using the Roche UPL Design Center (Roche Diagnosis GmbH, Mannheim, Germany) and are listed in Additional file 2: Table 3. Data acquisition and raw data analysis were performed using QuantStudio PCR Systems (Applied Biosystems). The Ct values of target genes were normalized to housekeeping genes (*GAPDH*, *ACTB and PUM1)* and analyzed with the ΔΔCt method [4].

To prepare cDNA for the subsequent quantification of miRNAs, miScript RT Kit (Qiagen, Hilden, Germany) was used according to the manufacturer's recommendations. For quantification of miRNAs, miScript PCR system (Qiagen, Hilden, Germany) was used. Raw data analysis was performed by using QuantStudio PCR Systems (Applied Biosystems). The Ct values of target genes were normalized to *SNORD72* and *SNORD95* and analyzed with the ΔΔCt method.

Protein isolation and Western blotting

Cells were seeded in 6-well plates for pre-treatment (miRNA mimics and siRNAs). After treatment, the cells were placed on ice and lysed with RIPA lysis buffer (Thermo Fisher Scientific, Massachusetts, USA) containing Complete Mini protease inhibitor cocktail and PhosSTOP phosphatase inhibitor (Roche Applied Science, Penzberg, Germany). The concentrations of protein samples were determined with BCA Protein Assays Kit (Thermo Fisher Scientific, Massachusetts, USA) and quantified with GloMax microplate reader (Promega GmbH, Walldorf, Germany).

Protein lysates (15-30 µg) were mixed with 4×RotiLoad protein loading buffer and denatured at 95°C for 5 min. Denatured protein samples were loaded on Mini-PROTEAN TGX Precast Gels and electrophoresis was performed at 145V for 75 min. The proteins were transferred from gels to PVDF membranes (Merck Millipore, Massachusetts, USA) by using the Trans-BlotR Turbo Transfer System (Bio-Rad, California, USA). The membranes were blocked with Rockland blocking buffer for 1 h

at room temperature on the shaker and subsequently incubated with a primary antibody diluted in blocking buffer overnight at 4°C on a rocking platform. After washed 3×10 min with 1×TBST, the membrane was incubated with fluorescent-labeled secondary antibody for 1 h at room temperature on the shaker and then washed 3×10 min with 1×TBST. The primary and secondary antibodies used in this study are listed in Supplementary Table 4. The membranes were scanned and probed using the Odyssey Infrared Imaging System (LI-COR Biosciences, Nebraska, USA). The signal intensity of the band was quantified by using ImageStudio software (LI-COR Biosciences, Nebraska, USA).

Mass spectrometry

Protein samples (10 µg per sample) were submitted to DKFZ Genomics and Proteomics Core Facility for mass spectrometry-based protein analysis. The samples were loaded on SDS-PAGE-gel to run for 0.5 cm. After Coomassie staining, the protein bands were cut out unfractionated and used for in-gel digestion on a DigestPro MSi robotic system (INTAVIS Bioanalytical Instruments) according to a modified protocol described by *Shevchenko et al* [5]. Peptides were loaded on a cartridge trap column, packed with Acclaim PepMap300 C18, 5 µm, 300 Å wide pore (Thermo Fisher Scientific, Massachusetts, USA) and separated in a three step, 180 min gradient from 3% to 40% ACN on a nanoEase MZ Peptide analytical column (300Å, 1.7 µm, 75 µm x 200 mm, Waters) carried out on a UltiMate 3000 UHPLC system. Eluting peptides were analyzed online by a coupled Q-Exactive-HF-X mass spectrometer (Thermo Fisher Scientific, Massachusetts, USA) running in data dependent acquisition mode where one full scan at 120 k resolution was followed by up to 35 MSMS scans at 15 k resolution of eluting peptides at an isolation window of 1.6 m/z and a collision energy of 27 NCE. Unassigned and singly charged peptides were excluded from fragmentation and dynamic exclusion was set to 60 sec to prevent oversampling of same peptides.

Raw data was analyzed by MaxQuant computational platform (version 1.6.3.3) using an organism-specific database extracted from Uniprot.org under default settings. Identification FDR cutoffs were 0.01 on peptide level and 0.01 on protein level. Match between runs option was enabled to transfer peptide identifications across raw files based on accurate retention time and m/z. Quantification was done by using a label-free quantification (LFQ) approach based on the MaxLFQ algorithm [6]. A minimum of two quantified peptides per protein was required for protein quantification. The Perseus software package (version 1.6.13.0) was used for imputation of missing values at default settings and statistical analysis [7]. Raw data were processed by in-house compiled R-scripts to plot and filter data. The analysis results were visualized in heatmaps. For the GSEA graphs, an adapted version of the replotGSEA function from the Rtoolbox was used (https://github.com/PeeperLab/Rtoolbox).

Luciferase reporter assay

Direct targets of a miRNA of interest were validated by 3'UTR dual luciferase reporter assay. The 3'UTR of *E2F1* was cloned into the luciferase reporter plasmid psiCHECK-2 harboring both firefly and renilla luciferase genes. Hence, cotransfection of the

luciferase reporter plasmid and a miRNA mimic directly targeting the 3'UTR under investigation results in a decrease in renilla luciferase activity, while the activity of the constitutively expressed firefly luciferase is used to normalize to exclude the differences caused by cell viability and transfection efficiency. For further validation, 4-sites mutated E2F1 3'UTR reporter plasmid is used to test whether the effect of the respective miRNA is abolished by mutation of the miRNA-binding sites. The sequences of primers for cloning are listed in Additional file 2: Table 2.

$1.2x10^4$ cells were seeded in white 96-well plates and transfected with miRNA mimics and 3'UTR reporter plasmid. After 48 h transfection, the cells were washed with 100 µL PBS and then lysed in 100 µL lysis buffer for 10 min at room temperature. 50 µL of the initial lysate were transferred to a new white 96-well plate for firefly luciferase activity measurement. The remaining lysate was used for renilla luciferase activity measurement. The plates were measured using GloMax Microplate Reader (Promega Gmbh, Walldorf, Germany). The composition of the buffer used in luciferase assay is listed in Additional file 2: Table 5.

1. Fellmann C, Hoffmann T, Sridhar V, Hopfgartner B, Muhar M, Roth M, Lai DY, Barbosa IA, Kwon JS, Guan Y *et al*: **An optimized microRNA backbone for effective single-copy RNAi**. *Cell reports* 2013, **5**(6):1704-1713.
2. Soneoka Y, Cannon PM, Ramsdale EE, Griffiths JC, Romano G, Kingsman SM, Kingsman AJ: **A transient three-plasmid expression system for the production of high titer retroviral vectors**. *Nucleic acids research* 1995, **23**(4):628-633.
3. Hartley JL, Temple GF, Brasch MA: **DNA cloning using in vitro site-specific recombination**. *Genome research* 2000, **10**(11):1788-1795.
4. Yuan JS, Reed A, Chen F, Stewart CN, Jr.: **Statistical analysis of real-time PCR data**. *BMC bioinformatics* 2006, **7**:85.
5. Shevchenko A, Tomas H, Havlis J, Olsen JV, Mann M: **In-gel digestion for mass spectrometric characterization of proteins and proteomes**. *Nature protocols* 2006, **1**(6):2856-2860.
6. Cox J, Hein MY, Luber CA, Paron I, Nagaraj N, Mann M: **Accurate proteome-wide label-free quantification by delayed normalization and maximal peptide ratio extraction, termed MaxLFQ**. *Molecular & cellular proteomics : MCP* 2014, **13**(9):2513-2526.
7. Tyanova S, Cox J: **Perseus: A Bioinformatics Platform for Integrative Analysis of Proteomics Data in Cancer Research**. *Methods in molecular biology* 2018, **1711**:133-148.
